# Thyroid Hormone Activates Brown Adipose Tissue and Increases Non-Shivering Thermogenesis - A Cohort Study in a Group of Thyroid Carcinoma Patients

**DOI:** 10.1371/journal.pone.0145049

**Published:** 2016-01-19

**Authors:** Evie P. M. Broeders, Guy H. E. J. Vijgen, Bas Havekes, Nicole D. Bouvy, Felix M. Mottaghy, Marleen Kars, Nicolaas C. Schaper, Patrick Schrauwen, Boudewijn Brans, Wouter D. van Marken Lichtenbelt

**Affiliations:** 1 MUMC+, Department of Human Biology, Maastricht, the Netherlands; 2 St. Franciscus Gasthuis, Department of Surgery, Rotterdam, the Netherlands; 3 MUMC+, Department of Nuclear Medicine, Maastricht, the Netherlands; 4 Department of Nuclear Medicine, University Hospital RWTH Aachen University, Aachen, Germany; 5 MUMC+, Department of Endocrinology, Maastricht, the Netherlands; 6 MUMC+, Department of General Surgery, Maastricht, the Netherlands; Pennington Biomedical Research Center, UNITED STATES

## Abstract

**Background/Objectives:**

Thyroid hormone receptors are present on brown adipose tissue (BAT), indicating a role for thyroid hormone in the regulation of BAT activation. The objective of this study was to examine the effect of thyroid hormone withdrawal followed by thyroid hormone in TSH-suppressive dosages, on energy expenditure and brown adipose tissue activity.

**Subjects/Methods:**

This study was a longitudinal study in an academic center, with a follow-up period of 6 months. Ten patients with well-differentiated thyroid carcinoma eligible for surgical treatment and subsequent radioactive iodine ablation therapy were studied in a hypothyroid state after thyroidectomy and in a subclinical hyperthyroid state (TSH-suppression according to treatment protocol). Paired two-tailed t-tests and linear regression analyses were used.

**Results:**

Basal metabolic rate (BMR) was significantly higher after treatment with synthetic thyroid hormone (levothyroxine) than in the hypothyroid state (BMR 3.8 ± 0.5 kJ/min versus 4.4 ± 0.6 kJ/min, *P* = 0.012), and non-shivering thermogenesis (NST) significantly increased from 15 ± 10% to 25 ± 6% (*P* = 0.009). Mean BAT activity was significantly higher in the subclinical hyperthyroid state than in the hypothyroid state (BAT standard uptake value (SUV^Mean^) 4.0 ± 2.9 versus 2.4 ± 1.8, *P* = 0.039).

**Conclusions:**

Our study shows that higher levels of thyroid hormone are associated with a higher level of cold-activated BAT.

**Trial Registration:**

ClinicalTrials.gov NCT02499471

## Introduction

Obesity has emerged to be the second leading cause of preventable death in Western society [1]. In recent years brown adipose tissue (BAT) received strong scientific interest due to its capacity to increase energy expenditure (EE).

Brown adipose tissue is suggested to be an important regulator of the energy balance [2,3]. The tissue is strongly innervated by the sympathetic nervous system. Where white adipose tissue (WAT) mainly stores energy, BAT has the capability to burn off excess calories by uncoupling the oxidation of substrate from ATP production via mitochondrial uncoupling protein 1 (UCP1), a process allowing the dissipation of energy as heat [4]. In response to cold exposure, BAT generates heat in order to prevent hypothermia. So far, cold-exposure seems to be the most potent stimulator for BAT thermogenesis [5–7].

The thyroid gland secretes thyroid hormone, which increases basal metabolic rate (BMR) in humans [8,9]. The effect of thyroid hormone on EE in humans is significant, as illustrated in clinical states of hypo- or hyperthyroidism where energy expenditure can decrease or increase up to three times compared to baseline [10]. The thyroid gland mainly secretes the inactive pro-hormone thyroxine (T4), which needs to be deiodinised in the target cells by deiodinases to the active hormone T3. Thyroxine (T4) supplementation has been suggested to increase resting metabolism [11]. Treatment of human preadipocytes with T3 stimulates the development of UCP-1-positive cells in white adipose tissue [12]. This suggests that thyroid hormone can also affect BAT.

In rodents, thyroid hormone-induced stimulation of BAT has been documented. After conversion of T4 to T3, thyroid hormone enhances BAT thermogenesis [13]. Thyroid-stimulating hormone (TSH), secreted by thyrotrope cells in the anterior pituitary gland, stimulates the thyroid gland to release T4. Interestingly, TSH is also involved in thermogenesis and TSH-receptors are present on brown adipocytes in rats [14]. This suggests that both TSH and T3 might be important in BAT function.

Besides the direct influence of thyroid hormone on the brown adipocyte, thyroid hormone could also be a central inducer of BAT by directly stimulating the hypothalamic pathway [15]. Systemic hyperthyroidism or central administration of thyroid hormone to rats has been shown to cause a decrease in hypothalamic AMP-activated protein kinase, leading to sympathetic activation and BAT induction.

Since BAT is known to become active during cold exposure, this suggests a possible link between thyroid hormone and cold-induced BAT activity [16]. Furthermore, a recent study showed induction of BAT D2 activity after cold acclimation in mice [17]. A recent case report of a thyroid carcinoma patient with extreme insulin resistance and thyroid cancer showed that therapeutic treatment with high doses of thyroid hormone was accompanied with active BAT on PET-CT, without cold exposure [18]. Besides this, a recent study in hyperthyroid patients showed a 3-fold higher glucose uptake in BAT compared with healthy subjects [19].

After complete resection of the thyroid gland, thyroid carcinoma patients will develop a hypothyroid state in order to be able to effectively eradicate possible thyroid remnants with radioactive iodine ablation therapy. The high levels of TSH will then maximally stimulate the thyroid remnant to incorporate iodine and thus increase the effect of the administered radioactive iodine dose. After administering the I^131^-therapy, thyroid hormone suppletion with levothyroxin is started in TSH-suppressive doses to decrease plasma TSH to levels < 0.1 mU/L. Since large changes in thyroid hormone levels are routinely observed in these patients this patient group offers the unique opportunity to observe the effect of hypothyroidism on physiological and metabolic parameters like blood pressure, skin temperature, BMR, NST (non-shivering thermogenesis) and BAT activity both in a hypothyroid and in the subsequent subclinical hyperthyroid state within the same subject. Therefore, we investigated the combined the effect of well-differentiated thyroid carcinoma treatment on several physiological and metabolic parameters, including BAT activity. So far, no studies have directly investigated the proposed synergism between thyroid hormone and cold-induced regulation of BAT activity.

## Materials and Methods

**Fig 1:**
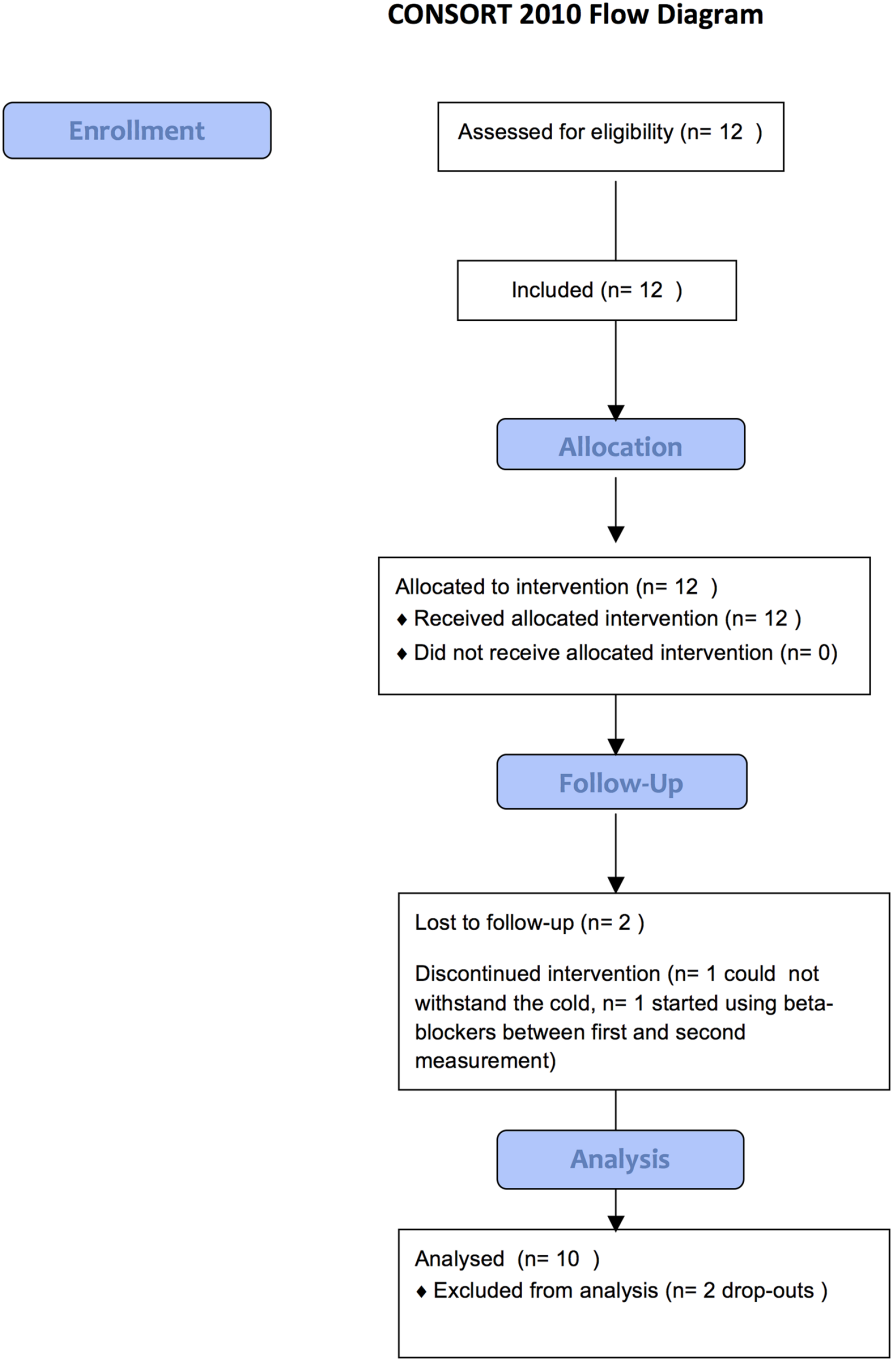
Consort 2010 flow chart.

The study was reviewed and approved of by the medical ethics committee of the Maastricht University Medical Centre (METC 11-3-081). This study was registered on ClinicalTrials.gov after enrollment of the subjects and in the Dutch CCMO register before enrollment of the subjects. The authors confirm that all ongoing and related trials for this drug/intervention are registered. Written informed consent was obtained from twelve subjects with well-differentiated thyroid carcinoma, two male and 10 female, who were treated and included in the study at Maastricht University Medical Center between June 2012 and July 2014. Unfortunately, there were two female dropouts during the study. One subject could not withstand the cold and one subject started using a beta-blocker between the first and second set of measurements (Fig 1). These subjects were excluded from the analyses.

To avoid interference of the menstrual cycle with the measurements, all female subjects were postmenopausal or used a specific oral contraceptive pill (ethinylestradiol/levonorgestrel 20 μg/100 μg) throughout the study. Subjects were screened before participation and all reported to have stable physical activity for at least six months (no participation in vigorous exercise program in the last six months, exercise maximum 2 times a week and maximum 3 hours a week). For compliance reasons, subjects who were psychologically unstable and subjects with mental retardation or severe behavior disorders were excluded from the study. Other exclusion criteria were pregnancy, the use of beta-blockers, and participation in an intensive weight-loss program during the last year before the start of the study, drugs and/or alcohol abuse and insulin-dependent type 2 diabetes or diabetes-related complications. All subjects were their own control, as they were all measured during the two conditions; hypothyroid state six to eight weeks after surgical removal of the thyroid gland and subclinical hyperthyroid state after administration of levothyroxine for four to six months.

### Study protocol

The first set of measurements took place between June 2012 and November 2013, on average 6.8 ± 2.8 weeks after surgery, when plasma free T4-levels were at the minimum. Subjects were measured in the morning after overnight fasting and asked to refrain from heavy exercise 24 hours before the measurements. During the measurements subjects were allowed to wear light standardized clothes (socks 0.02 clo, shirt with long sleeves 0.20 clo, sweatpants 0.28 clo, underwear 0.04 clo, total clo factor 0.54 clo). Clo units refer to clothing insulation [20].

**Fig 2:**
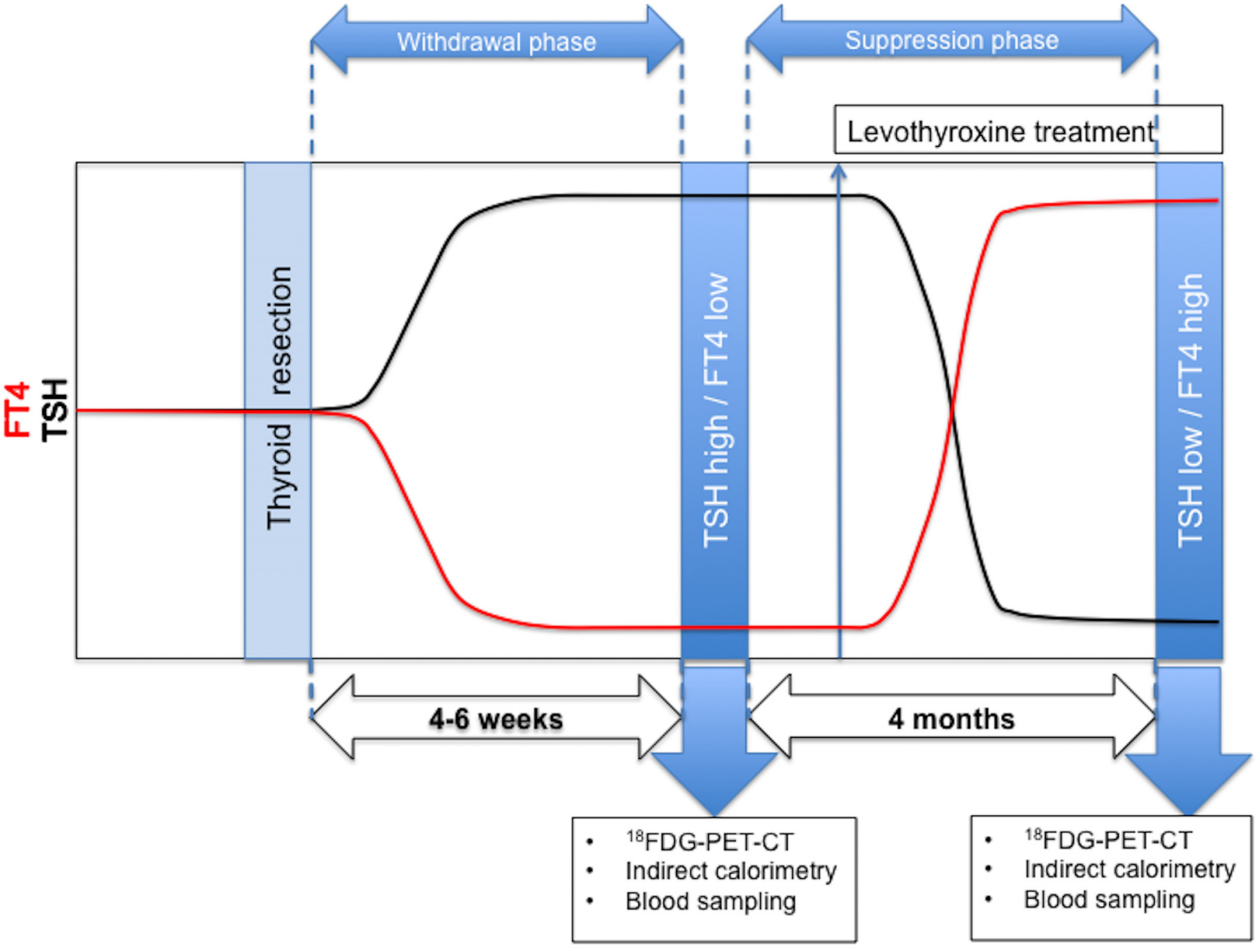
Schematic representation of study measurements after total thyroidectomy in the Maastricht University Medical Centre. Blue arrows indicate moment of study measurements. FT4 indicates free thyroxine, TSH indicates thyroid-stimulating hormone, levothyroxine treatment indicates pharmacological levothyroxine suppletion that suppresses endogenous TSH. I^131^ indicates radioactive iodine, used for radioactive ablation therapy of thyroid gland remnants after thyroid gland resection for well-differentiated thyroid carcinoma. I^124^ indicates a proton-rich isotope of iodine used as a radiochemical for determination of thyroid gland remnants after thyroid gland resection for well-differentiated thyroid carcinoma.

First, the subjects’ body composition was determined using dual x-ray absorptiometry (DXA, Hologic, type Discovery A, Bedford, MA). For the determination of body core temperature and heart rate, a telemetric pill (CoreTemp, HQ Inc., Palmetto, FL USA) was orally ingested by the study subjects. This measurement failed in three subjects. Skin temperature was determined by applying wireless iButtons on 14 ISO-defined sites of the body [21]. The second set of measurements were done four to six months after radioactive iodine ablation therapy, when the subjects were in the subclinical hyperthyroid state, on levothyroxine therapy, with low TSH and high fT4 levels (Fig 2). For detailed description see S1 Text—Study Protocol.

### Personal cooling protocol and ^18^F-FDG-PET-CT

Subjects were placed inside a specially equipped air permeable climate tent (Colorade Altitude Training, Louisville, CO). Inside this tent the air temperature was regulated by an air conditioner to maintain the air temperature within the tent with an accuracy of 1°C [22]. To guarantee a comfortable position during the measurements, subjects were placed in a semi-supine position in a nephrodialysis chair. Subjects underwent a personalized cooling protocol. The seat of the nephrodialysis chair in which the subjects were placed was covered with a water-perfused matrass (Blankett role, Cincinatti sub zero 2000, USA), allowing for extra cooling options for the dorsal side of the body. To guarantee maximum non-shivering thermogenesis, we used a personalized cooling protocol described earlier [7]. NST was defined as percentage increase in EE after cold exposure.

Energy expenditure was measured by indirect calorimetry during the three hours of the measurements. During the first hour, measurements were performed in thermoneutrality (room temperatures 23.7 ± 0.85°C in hypothyroid state, 23.8 ± 0.26°C in TSH-suppressed state), followed by a gradual step-wise decrease of the temperature of the air and cooling mattress during the second hour. This was continued until the onset of shivering. Shivering was detected visually and subjects were asked to report shivering on a visual analog scale every ten minutes, as described in earlier studies by our group [23]. At the first signs of shivering, air and water temperatures were increased by steps of 1°C until shivering just stopped and temperatures were then kept stable at these points. After the first hour of cold exposure 75 MBq of ^18^-Fluoro-Deoxy-Glucose (^18^F-FDG) was injected through a intravenous catheter. After injection, cold-exposure was maintained for another hour, in which subjects were instructed to lay still to prevent uptake of ^18^F-FDG in muscle tissue. Blood was withdrawn from the cubital vein catheter once during thermoneutrality (55 minutes after the start of the protocol and right before the onset of cooling) and once during cold exposure (115 minutes after the start of the protocol and right before the ^18^F-FDG injection).

After the third hour, subjects were moved to the positron emission and computed tomography scanner (PET-CT-scanner) (Gemini TF PET-CT, Philips, The Netherlands) for quantification of metabolically active BAT. The scanning protocol and data interpretation methods were identical to those used in earlier studies by our group [2]. For detailed description see S1 Text—Study Protocol.

### Follow-up

All patients underwent their thyroidectomy and radioactive iodine ablation therapy, according to treatment protocol, without any complications. The second set of measurements took place between October 2012 and July 2014, four to six months after the initial measurements, after subjects were stable on a daily dose of synthetic thyroid hormone (levothyroxine, fT4 levels 23.1 ± 3.9 pmol/L, TSH 0.5 ± 0.6 mU/L levothyroxine dose 137.75 ± 23.75 μg/day), the above described measurement protocol was repeated.

### Blood analyses

Blood was collected from the antecubital vein for analyses of several blood variables. Plasma concentrations of free fatty acids (NEFA-HR set; Wako Chemicals), free glycerol (Glycerol kit; R-Biopharm), total glycerol (ABX Triglyceriden CP; Horiba ABX), and glucose (ABX Glucose HK CP; Horiba ABX) were measured on a COBAS PENTRA centrifugal spectrophotometer (Horiba ABX). Plasma triglyceride concentrations were calculated by subtracting free glycerol from the total glycerol concentrations, and serum insulin was analyzed on a Gamma Counter (2470 Automatic Gamma Counter Wizard2 Wallac; Perkin-Elmer) with a Human Insulin specific RIA kit (Millipore). Plasma norepinephrine and adrenaline were analyzed by using reagents from Recipe Chemicals and Instruments with HPLC through electrochemical detection. Serum TSH was measured by an Electrochemiluminescence Immunoassay Kit on a COBAS 6000 system (Roche Diagnostica), and free thyroxine (FT4) was analyzed by a solid-phase time-resolved fluoroimmunoassay kit on an AutoDELFIA system (PerkinElmer). Plasma inflammatory marker C- reactive protein (CRP) was measured with a particle-enhanced immunoturbidimetric assay on a COBAS PENTRA system (Horiba ABX). For detailed description see S1 Text—Study Protocol.

### Statistical analysis

Statistical analysis was performed by PASW Statistics version 20.0 for MacBook Pro.

Unfortunately, no studies have been performed with thyroid hormone in which our most important outcome parameters (BAT activity) is studied. Still, we decided to base our power calculation on the SUV of BAT. A previous study from our group showed an increase of BAT activity in lean subjects upon 10 days of cold acclimation from 2.4 standard uptake value (SUV) mean to 2.8 SUV mean, with a standard deviation of 0.5 (31). In the current study, in which slightly older subjects are included, we expect BAT values to be lower.

We expect a slightly bigger increase in BAT activity as compared to 10 days of mild cold exposure. Therefore, we expect BAT activity values to be 1.4 SUV mean in the hypothyroid state and 1.85 SUV mean in the subclinical hyperthyroid state, with a standard deviation of 0.5. With this information, a two-sided alpha of 0.05, and a power of 0.80, our study group should consist of 10 participants (calculated using G*Power 3.1 software, Faul, Erdfelder, Land and Buchner, University of Trier). Accounting for a possible dropout rate of 15%, a total of (10/0.85 =) 12 subjects were included. In the end, two subjects dropped out.

Reported data is expressed as means of ±SD. Total BAT activity was expressed in standard uptake values (SUV; as calculated by uptake (kilobequerels per milliliter) per injected dose (kilobequerels) per patient weight (grams)). BAT activity of each region was determined by the average SUV (SUV mean) times the volume of the region (cubic centimeter), expressed as SUV total. Paired two-tailed t-tests were used in order to compare data before and after cold exposure and data before and after treatment. Linear regression analyses were used to identify correlations between variables. For detailed description see S1 Text—Study Protocol.

### Subject characteristics

This study included eight female and two male patients with well-differentiated thyroid carcinoma that were selected for total thyroidectomy according to national and local oncology guidelines as defined in the Dutch oncology guidelines for thyroid carcinoma [24]. Subject characteristics are shown in Table 1. Surgical removal of the thyroid gland was on average 6.8 ± 2.8 weeks before the first set of measurements and subjects did not have any adjustments to their levothyroxine medication (last adjustment > one month before the second set of measurements). The first set of measurements took place on the same day as the radioactive iodine ablation therapy, after which levothyroxine therapy was started. On average, subjects were remeasured 4.2 ± 1.4 months after the first measurement, and average dosage of levothyroxine at the time of the second set of measurements was 137.75 ± 19.75 μg/day.

**Table 1:** Subject characteristics (N = 10).

	Before	After	P-value
Age (yr)	47.4 ± 12.0	47.7 ± 12.0	0.081
BMI (kg/m2)	30.2 ± 7.0	30.1 ± 6.6	0.917
Body mass (kg)	82.3 ± 15.1	83.5 ± 17.3	0.340
Body fat (%)	33.8 ± 10.8	33.9 ± 8.5	0.920
Fat mass (kg)	27.7 ± 10.5	29.5 ± 11.8	0.024 *
Fat free mass (kg)	52.9 ± 7.5	52.5 ± 7.8	0.623
Free T4 (Umol/l)	5.6 ± 6.9	23.1 ± 3.9	< 0.001 **
TSH (Umol/l)	107.6 ± 55.9	0.5 ± 0.6	< 0.001 **
Levothyroxin dose (μg/day)	0	137.75 ± 19.75	

Subject characteristics in ten patients (two male, eight female) with measurements in the hypothyroid and euthyroid phase of thyroid carcinoma treatment. BMI indicates Body Mass Index. Values are expressed as means ± SD.

* *P* < 0.05;

** *P* < 0.01.

## Results

### Energy expenditure

**Fig 3:**
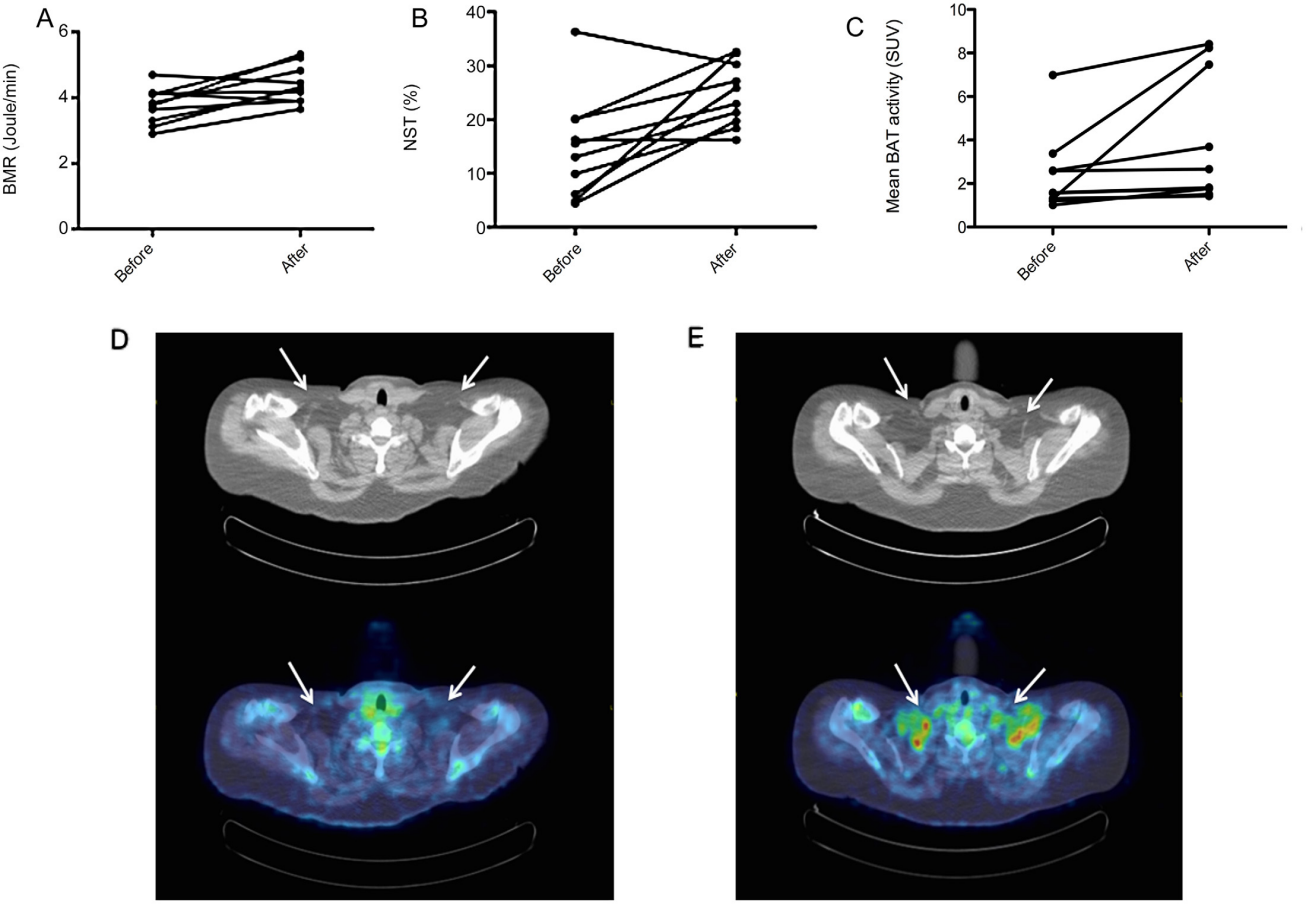
BAT activity, BMR and NST before and after levothyroxine therapy. Brown adipose tissue (BAT) activity before and after levothyroxine substitution therapy. (A) Basal metabolic rate (BMR) in joules per minute. (B) Non-shivering thermogenesis (NST) before and after levothyroxine replacement therapy. (C) BAT activity in Mean Standard Uptake Values (SUV mean) before and after levothyroxine therapy. Subject indicated with ^X^ is also depicted in Fig 2D and 2E. (D) Transversal CT (top) and PET/CT fusion (bottom) slice of the supraclavicular region demonstrating ^18^F-FDG-uptake in BAT locations (white arrows) after cold exposure in hypothyroid state. (E) Transversal CT (top) and PET/CT fusion (bottom) slice of the supraclavicular region demonstrating ^18^F-FDG-uptake in BAT locations (white arrows) after cold exposure in subclinical hyperthyroid state.

Basal metabolic rate significantly increased in the subclinical hyperthyroid state versus the hypothyroid state (4.4 ± 0.6 kJ/min versus 3.8 ± 0.5 kJ/min, *P* = 0.012, Fig 3A, Table 2). Furthermore, EE during cold exposure significantly increased after thyroid substitution therapy (4.3 ± 0.7 J/min versus 5.4 ± 0.5 J/min, *P* = 0.006). NST, also increased significantly in the presence of thyroid hormone (15 ± 10% versus 25 ± 6%, *P* = 0.009, Fig 3B).

**Table 2:** Skin perfusion, blood pressure, and body temperature under thermoneutral conditions and during mild cold exposure, before and after levothyroxine substitution (N = 10.)

	Before	After	P-value
**Mean skin temperature (°C)**			
Thermoneutral	32.2 ± 0.3	33.3 ± 0.4	< 0.001 **
Mild cold	29.5 ± 0.6	30.9 ± 0.7	0.001 **
Change upon cold stimulation	-2.6 ± 0.5	-2.5 ± 0.7	0.489
**Core temperature (°C)**			
Thermoneutral	37.1 ± 0.5	37.3 ± 0.6	0.633
Mild Cold	37.4 ± 0.4	37.2 ± 0.3	0.518
Change upon cold stimulation	0.3 ± 0.5	1.0 ± 0.8	0.517
**Gradient core—mean skin (°C)**			
Thermoneutral	5.0 ± 0.5	7.9 ± 0.7	0.173
Mild cold	4.2 ± 1.1	7.1 ± 1.4	0.325
Change upon cold stimulation	0.9 ± 3.7	3.3 ± 1.2	0.099
**Normalized skin perfusion hand (%)**			
Thermoneutral	100	100	
Mild cold	45 ± 21	62 ± 66	0.503
Change upon cold stimulation	-56 ± 22	-37 ± 45	0.462
**Systolic blood pressure (mmHg)**			
Thermoneutral	131 ± 23	124 ± 18	0.096
Mild cold	142 ± 27	130 ± 24	0.041 *
Change upon cold stimulation	11 ± 12	7 ± 11	0.159
**Diastolic blood pressure (mmHg)**			
Thermoneutral	93 ± 18	82 ± 15	0.004 **
Mild cold	98 ± 15	85 ± 15	0.006 **
Change upon cold stimulation	5 ± 9	3 ± 5	0.692
**Mean arterial pressure (mmHg)**			
Thermoneutral	105 ± 20	96 ± 18	0.013 *
Mild cold	113 ± 18	100 ± 18	0.009 **
Change upon cold stimulation	7 ± 9	3 ± 7	0.435
**Heart rate (BPM)**			
Thermoneutral	57 ± 18	67 ± 9	0.274
Mild cold	63 ± 9	66 ± 11	0.204
Change upon cold stimulation	6 ± 10	-1 ± 3	0.479
**Free fatty acids (μM)**	734 ± 166	531 ± 177	0.042 *
**Total glycerol (μM/L)**	1554 ± 291	976 ± 486	0.001 **
**CRP**	2.9 ± 4.5	4.8 ± 5.9	0.021 *
**Glucose (mM)**	5.0 ± 0.6	5.3 ± 0.4	0.024 *
**Insulin (μU/ml)**	7.9 ± 2.5	9.4 ± 5.0	0.220
**Noradrenalin** (N = 9)			
Thermoneutral	4.7 ± 3.8	2.2 ± 1.2	0.067
Mild cold	6.1 ± 3.0	3.6 ± 1.7	0.001 **
Change upon cold stimulation	1.5 ± 4.7	1.5 ± 0.8	0.979
**Adrenalin (N = 9)**			
Thermoneutral	0.2 ± 0.1	0.1 ± 0.1	0.116
Mild cold	0.2 ± 0.1	0.1 ± 0.1	0.007 **
Change upon cold stimulation	0 ± 0.2	0 ± 0.1	0.963

Values are expressed as means ± SD.

* *P* < 0.05;

** *P* < 0.01.

### BAT activity

All subjects (N = 10) had active cold-stimulated BAT, both in the hypothyroid and subclinical hyperthyroid state. Representative images of FDG-uptake on PET-CT in the studied group are shown in Fig 3. The mean SUV was higher in the presence of thyroid hormone in 9 of the 10 subjects. This increase was significant (BAT SUV mean; 4.0 ± 2.9 versus 2.4 ± 1.8, *P* = 0.039, Fig 3C, 3D and 3E). No correlations were found between changes in fT4 levels and changes in BAT activity (BAT SUVmean: r = -0.100, *P* = 0.783). Also, changes in TSH levels did not correlate with changes in BAT activity (BAT SUV mean: r = -0.122, *P* = 0.754). Changes in fT4 levels (delta fT4) were defined as: fT4 level in hypothyroid state minus fT4 level in subclinical hyperthyroid state. Changes in TSH levels and BAT activity were defined in a similar manner.

### Core and skin temperature

Body core temperature in thermoneutral and cold conditions did not significantly change in the presence of thyroid hormone (core temperature thermoneutral 37.1 ± 0.5°C versus 37.3 ± 0.6°C, *P* = 0.633; core temperature cold 37.4 ± 0.4°C versus 37.2 ± 0.3°C, *P* = 0.518, Table 2). Mean skin temperature in thermoneutral and cold conditions was significantly lower in the hypothyroid state (mean skin temperature thermoneutral 32.2 ± 0.3°C versus 33.3 ± 0.4°C, *P* < 0.001; mean skin temperature cold 29.5 ± 0.6°C versus 30.9 ± 0.7°C *P* = 0.001, Table 2). No correlations were found between mean skin temperature during cold exposure and BAT activity in both the hypothyroid and subclinical hyperthyroid state (r = -0.161, *P* = 0.656 and r = 0.035, *P* = 0.925 respectively).

### Skin perfusion and blood pressure

Hand skin blood flow was significantly reduced during cold exposure (hand; -47% ± 48%, *P* = 0.048, Table 2). There were no differences in the extent of skin blood flow reduction between the hypothyroid and subclinical hyperthyroid situation.

Diastolic blood pressure in the thermoneutral condition was significantly higher in the hypothyroid state (93 ± 18 versus 82 ± 15, *P* = 0.004, Table 2). Also, mean arterial pressure (MAP) in both thermoneutral and mild cold conditions was significantly higher in the hypothyroid state (105 ± 20 versus 96 ± 18, *P* = 0.013 and 113 ± 18 versus 100 ± 18, P = 0.009 respectively, Table 2).

### Blood values

Baseline free fatty acids and total glycerol were significantly lower in the subclinical hyperthyroid state than in the hypothyroid state (*P* = 0.042 and *P* = 0.001 respectively, Table 2), whereas baseline CRP and glucose levels were significantly higher in the clinical hyperthyroid state (*P* = 0.021 and *P* = 0.024 respectively, Table 2). During cold exposure, both noradrenaline and adrenaline levels were significantly lower in the subclinical hyperthyroid state than in the hypothyroid situation (*P* = 0.001 and *P* = 0.007 respectively, Table 2). In our study, changes in TSH levels (deltaTSH) did not correlate with changes in total glycerol (r = 0.429, *P* = 0.250) or changes in free fatty acid levels (r = -0.039, *P* = 0.921).

For full study data see S1 Table—Dataset.

## Discussion and Conclusion

In this study we showed that BAT activity significantly increased in the subclinical hyperthyroid state compared to the hypothyroid state after thyroid carcinoma treatment. The data also demonstrated the presence of functional BAT in the hypothyroid group. Furthermore, we showed that the increase in BAT activity was accompanied by a 26% increase in NST.

### Effects on BMR, NST and BAT activity

It has long been accepted that one of the main functions of thyroid hormone is homeostasis of overall EE and BMR to the benefit of growth and development [25]. Thyroidectomized rats have 20–30% reduction in BMR, which is immediately restored upon T4 injection [26]. Here, we showed that BMR significantly increased in the presence of thyroid hormone, when compared to the hypothyroid state.

It is well known that BAT thermogenesis is activated by the hypothalamus via the sympathetic nervous system (SNS) [27]. Here we showed a significant cold-stimulated BAT activation in hypothyroid patients, which is in line with a recent case report, which showed active BAT in a case of severe hypothyroidism [28]. Furthermore, a significant increase in BAT activity accompanied by an increased NST was detected after thyroid hormone substitution therapy. Changes in T4 and TSH values were not correlated with changes in BAT activity. The positive effect of high-dose levothyroxine treatment on BAT activity after treatment of thyroid carcinoma with total thyroidectomy and subsequent radioactive iodine ablation therapy was previously shown in a case report. However, without levothyroxine no BAT activity was detected in that patient [18]. The additive effect of thyroid hormone and cold-exposure on BAT activity found in this study could possibly be attributed to the up-regulation of beta-adrenergic receptors in response to thyroid hormone [29], leading to increased sensitivity to catecholamines.

### Body composition and temperatures

In our study, we found that in the hypothyroid state, average skin temperature at thermoneutrality was significantly lower than in the subclinical hyperthyroid state. Hypothyroidism patients often report cold extremities and cold intolerance ^37, personal observations^. The increase in mean skin temperature upon thyroid hormone replacement is probably accounted for by thyroid-induced modulation of metabolic rate. Also, skin temperatures during cold exposure were significantly higher in the clinical hyperthyroid state than in the hypothyroid state, although no correlations between average skin temperature during cold exposure and BAT activity were found. The reduction of thermogenesis seen in hypothyroidism has been known to be partially compensated by cutaneous vasoconstriction [30,31]. The decreased skin temperature seen in the hypothyroid situation could be this thyroid-driven vasoconstriction, for the benefit of internal heat preservation. However, in the absence of thyroid hormone, EE still increased and functionally active BAT was still observed upon cold exposure, as was expected from animal studies [32]. Interestingly, hypothyroid noradrenalin and adrenalin levels during mild cold exposure where significantly higher than those in the subclinical hyperthyroid state, which could indicate compensatory adrenergic activation. Systemic hyperthyroidism or central administration of thyroid hormone to rats has been shown to cause a decrease in hypothalamic AMP-activated protein kinase, leading to sympathetic activation and BAT induction. BAT can also be activated in the case of hypothyroidism. Exposing D2-deficient mice to a cold environment still leads to thermogenesis via activation of the sympathetic nervous system, despite the limited availability of active plasma thyroid hormone. However, when these animals are ot exposed to cold, they become glucose intolerant and develop non-alcoholic fatty liver disease and diet-induced obesity [32], suggesting the synergism between the sympathetic nervous system and thyroid axis in the control of BAT-mediated thermogenesis. The additive effect of thyroid hormone and cold-exposure on BAT activity could also be attributed to the up-regulation of beta-adrenergic receptors in response to thyroid hormone [29], leading to increased sensitivity to catecholamines. In hypothyroidism, responses to adrenergic stimulation are relatively low or blunted, leading to cold intolerance and limited metabolic response to cold exposure [33]. Possibly the significant cold-induced increase in catecholamines in the hypothyroid state is a feedback mechanism to the benefit of heat production.

### Metabolic health effects

In this study, during the hypothyroid state, blood pressure was significantly increased. Hypothyroidism has been known to have profound effects on multiple organs and organ systems. Lately, increased risk for cardiovascular disease due to hypothyroidism has received strong scientific attention. In this study we found that thyroid hormone substitution therapy has a positive effect on serum free fatty acids and total glycerol, alongside an improvement of systolic and diastolic blood pressure. A positive association between TSH levels and systolic and diastolic blood pressure has been described earlier [34]. In another publication by Liu et al. the prevalence of hypertension (defined as systolic blood pressure ≥ 140 mmHg and/or diastolic blood pressure ≥ 90 mmHg) was significant higher in a group with subclinical hypothyroidism (TSH > 4.8 mIU^-1^) than in the euthyroid group (TSH 0.3–4.8 mIU^-1^) [35]. Another possible explanation for the elevated blood pressure in hypothyroidism observed is the compensatory adrenergic activation (see above).

Recently thyroid- stimulating hormone has been identified as an inducer for lipolysis [36]. In our study we found significantly higher serum free fatty acids in the hypothyroid state (with high TSH, Table 2), alongside increased serum total glycerol (Table 2), suggesting a role for TSH in metabolic health. This finding is in accordance with earlier studies, in which recombinant TSH was administered to 19 patients with differentiated thyroid carcinoma, who had previously undergone surgical thyroidectomy with consecutive radioactive iodine ablation therapy. In this study a 42% increase in FFAs was found [37]. In our study, no correlations were found between TSH, total glycerol and FFA levels. The higher levels of total glycerol observed in the hypothyroid state could result from lower BAT activation in this state, since BAT is known to control triglyceride clearance [38].

The major limitations in this study are the small number of subjects enrolled. However, the used protocol, which allowed us to study the same subject in the hypothyroid as well as the subclinical hyperthyroid state, increased the power of the study. Taken together, in the present study we showed that higher levels of thyroid hormone positively affect BAT activity, BMR and NST. This is in line with the postulated thyroid hormone and sympatho-adrenal synergy. This suggests that thyroid hormone is an effective activator for brown adipose tissue and cold-induced thermogenesis. If safe, BAT-targeted thyroid thermogenesis could increase energy expenditure [39]. However, since this study consisted of measurements in a group of cancer patients, more studies regarding the effects of thyroid hormone on BAT activity and energy metabolism in healthy subjects are warranted and detailed data on possible side effects is needed.

## Supporting Information

S1 Checklist TREND Checklist. (PDF)

S1 Table Dataset. (PDF)

S1 Text Study Protocol. (PDF)
