# Correction: Thyroid Hormone Activates Brown Adipose Tissue and Increases Non-Shivering Thermogenesis—A Cohort Study in a Group of Thyroid Carcinoma Patients

**DOI:** 10.1371/journal.pone.0209225

**Published:** 2018-12-12

**Authors:** Evie P. M. Broeders, Guy H. E. J. Vijgen, Bas Havekes, Nicole D. Bouvy, Felix M. Mottaghy, Marleen Kars, Nicolaas C. Schaper, Patrick Schrauwen, Boudewijn Brans, Wouter D. van Marken Lichtenbelt

During a reanalysis of the study, the authors discovered several inaccuracies in the data. Therefore, the authors recalculated the complete dataset and discovered additional errors, detailed below.

There is an error in the first sentence of the Study protocol subsection of the Materials and Methods section. The correct sentence is: The first set of measurements took place between June 2012 and November 2013, on average 6.8 ± 3.2 weeks after surgery, when plasma free T4-levels were at the minimum.

There is an error in the caption for Fig 2. Please see the complete, correct Fig 2 caption here.

**Fig 2:**
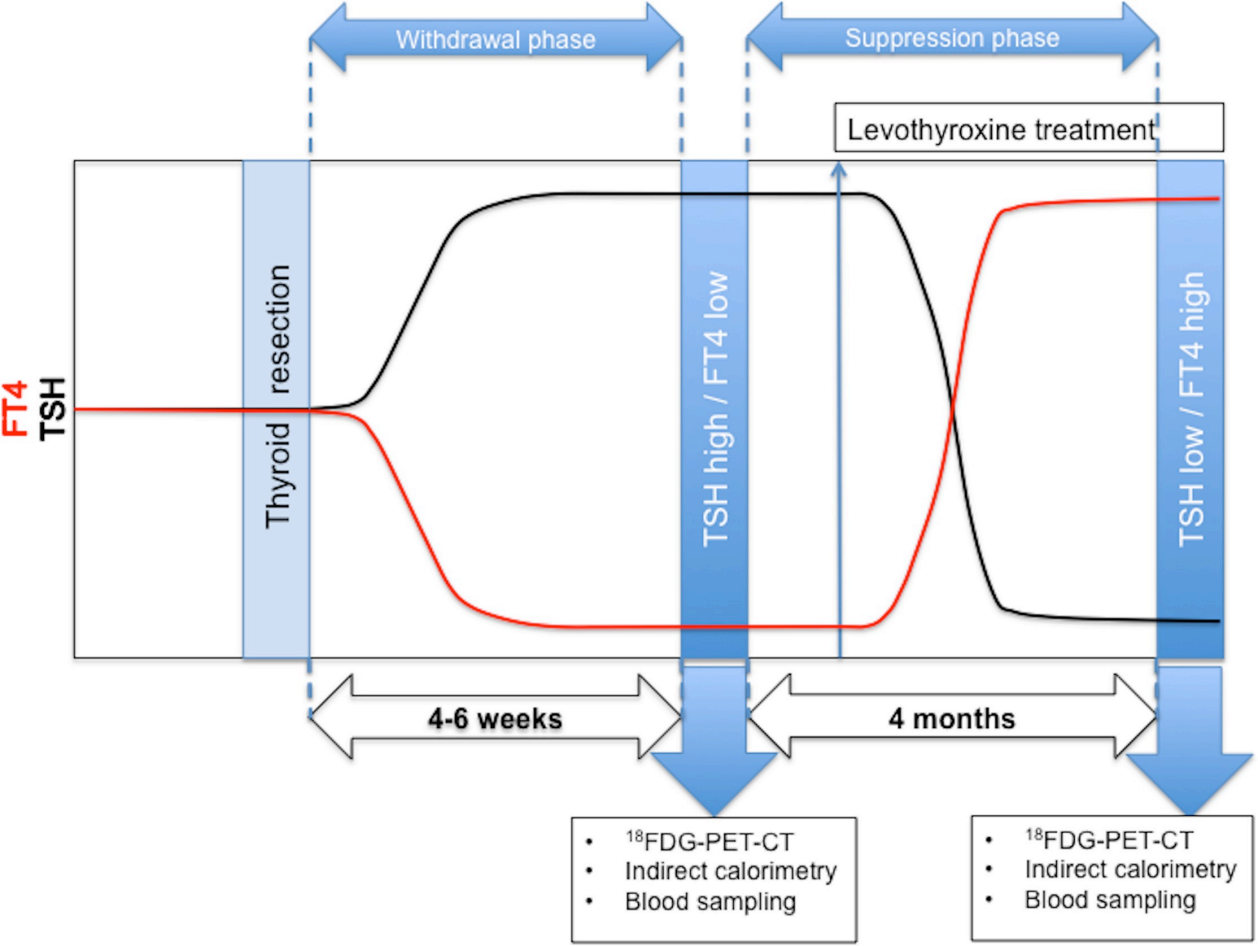
Schematic representation of study measurements after total thyroidectomy in the Maastricht University Medical Centre. Blue arrows indicate moment of study measurements. FT4 indicates free thyroxine, TSH indicates thyroid-stimulating hormone, levothyroxine treatment indicates pharmacological levothyroxine suppletion that suppresses endogenous TSH.

There is an error in the second sentence of the Follow-up subsection of the Materials and Methods section. The correct sentence is: The second set of measurements took place between October 2012 and July 2014, four to six months after the initial measurements, after subjects were stable on a daily dose of synthetic thyroid hormone (levothyroxine, fT4 levels 23.1 ± 3.9 pmol/L, TSH 0.5 ± 0.6 mU/L levothyroxine dose 143.75 ± 23.75 μg/day), the above described measurement protocol was repeated.

There are errors in the final sentence of the Subject characteristics subsection of the Materials and Methods section. The correct sentence is: On average, subjects were remeasured 5.2 ± 1.8 months after the first measurement, and average dosage of levothyroxine at the time of the second set of measurements was 143.75 ± 23.75 μg/day.

There are errors in the final sentence of the Energy expenditure subsection of the Results section. The correct sentence is: NST, also increased significantly in the presence of thyroid hormone (15 ± 10% versus 23.3 ± 5.4%, *P* = 0.005, Fig 3B).

There are errors in the third sentence of the BAT activity subsection of the Results section. The correct sentence is: This increase was just not significant (BAT SUV mean; 3.88 ± 2.96 versus 2.4 ± 1.8, *P* = 0.053, Fig 3C, 3D and 3E).

There are errors in the first sentence of the Core and skin temperature subsection of the Results section. The correct sentence is: Body core temperature in thermoneutral and cold conditions did not significantly change in the presence of thyroid hormone (core temperature thermoneutral 37.1 ± 0.4°C versus 37.3 ± 1.1°C, *P* = 0.633; core temperature cold 37.3 ± 0.5°C versus 37.7 ± 1.7°C, *P* = 0.538, Table 2).

There are errors in the first sentence of the Skin perfusion and blood pressure subsection of the Results section. The correct sentence is: Hand skin blood flow was significantly reduced during cold exposure (hand: -59 ± 29% during hypothyroidism versus -35 ± 64% after levothyroxine treatment, *P* = 0.048, Table 2).

There are errors in the fourth sentence of the Skin perfusion and blood pressure subsection of the Results section. The correct sentence is: Also, mean arterial pressure (MAP) in both thermoneutral and mild cold conditions was significantly higher in the hypothyroid state (105 ± 20 mmHg versus 96 ± 16 mmHg, *P* = 0.008 and 113 ± 19 mmHg versus 100 ± 16 mmHg, *P* = 0.010, Table 2).

There are errors in the Blood values subsection of the Results section. The correct first three sentences are: Total glycerol was significantly lower in the subclinical hyperthyroid state than in the hypothyroid state (*P* = 0.001). A similar trend was seen for free fatty acids (*P* = 0.083).

There are errors in the second sentence of the Blood values subsection of the Results section. The correct sentence is: During cold exposure, both noradrenaline and adrenaline levels were significantly lower in the subclinical hyperthyroid state than in the hypothyroid situation (*P* = 0.001 and *P* = 0.011 respectively, Table 2).

There are errors in Table 1. Please see the corrected Table 1 here.

**Table 1:** Subject characteristics (n = 10).

	Before	After	P-value
Age (yr)	47.6 ± 10	48.2 ± 10	0.005
BMI (kg/m^2^)	29.1 ± 5.8	29.6 ± 6.6	0.456
Body mass (kg)	82.3 ± 15.2	83.5 ± 17.3	0.478
Body fat (%)	32.5 ± 8.4	33.9 ± 8.5	0.011
Fat mass (kg)	27.7 ± 10.5	29.5 ± 11.8	0.024
Soft Fat free mass (kg)	52.9 ± 7.5	52.5 ± 7.8	0.623
fT4 (pmol/L)	3.4 ± 0.8	23.1 ± 3.9	<0.001
TSH (mU/L)	104.9 ± 53.6	0.5 ± 0.6	<0.001
Levothyroxin dose (μg/day)	NA	143.8 ± 23.8	NA

Subject characteristics in ten patients (two male, eight female) with measurements in the hypothyroid and euthyroid phase of thyroid carcinoma treatment. BMI indicates Body Mass Index. Values are expressed as means ± SD.

* P < 0.05

** P < 0.01.

There are errors in Table 2. Please see the corrected Table 2 here.

**Table 2:** Body temperature, skin perfusion, blood pressure and catecholamines under thermoneutral conditions and during mild cold exposure, before and after levothyroxine substitution. Blood values under thermoneutral conditions before and after levothyroxine substitution.

	Before	After	P-value
**Mean skin temperature (°C)**			
Thermoneutral	32.2 ± 0.3	33.3 ± 0.4	**<0.001**
Mild cold	29.5 ± 0.6	30.9 ± 0.7	**0.001**
Change upon cold stimulation	-2.7 ± 0.5	-2.4 ± 0.8	0.338
Core temperature (°C)*			
Thermoneutral	37.1 ± 0.4	37.3 ± 1.1	0.633
Mild cold	37.3 ± 0.5	37.7 ± 1.7	0.538
Change upon cold stimulation	0.1 ± 0.1	0.4 ± 0.6	0.340
Gradient core–mean skin (°C)*			
Thermoneutral	5.0 ± 0.5	4.1 ± 1.1	0.173
Mild cold	7.7 ± 0.5	6.7 ± 1.4	0.186
Change upon cold stimulation	2.7 ± 0.3	2.6 ± 0.7	0.394
Normalized skin perfusion hand (%)*			
Thermoneutral	100	100	NA
Mild cold	41 ± 29	64 ± 64	0.469
Change upon cold stimulation	59 ± 29	35 ± 64	0.469
Systolic blood pressure (mmHg)**			
Thermoneutral	131± 23	124 ± 18	0.092
Mild cold	143 ± 27	130 ± 24	**0.034**
Change upon cold stimulation	12 ± 11	7 ± 11	0.356
Diastolic blood pressure (mmHg)**			
Thermoneutral	92 ± 18	82 ± 15	**0.004**
Mild cold	98 ± 15	85 ± 17	**0.007**
Change upon cold stimulation	5 ± 9	3 ± 5	0.629
MAP (mmHg)**			
Thermoneutral	105 ± 20	96 ± 16	**0.008**
Mild cold	113 ± 19	100 ± 16	**0.010**
Change upon cold stimulation	7 ± 9	4 ± 5	0.479
Heart rate (bpm)***			
Thermoneutral	63 ± 7	69 ± 7	0.099
Mild cold	63 ± 8	67 ± 9	0.204
Change upon cold stimulation	-0 ± 2	-2 ± 3	0.916
**Noradrenalin (nmol/L)**			
Thermoneutral	4.5 ± 3.6	2.1 ± 1.2	**0.047**
Mild cold	6.2 ± 2.9	3.5 ± 1.7	**0.001**
Change upon cold stimulation	1.7 ± 4.2	1.4 ± 0.8	0.786
**Adrenalin (nmol/L)**			
Thermoneutral	0.21 ± 0.08	0.13 ± 0.08	0.086
Mild cold	0.19 ± 0.12	0.09 ± 0.04	**0.011**
Change upon cold stimulation	-0.02 ± 0.15	-0.04 ± 0.07	0.749
**Free fatty acids (μmol/L)**	733.9 ± 165.8	561.8 ± 172.4	0.083
**Total glycerol (μmol/L)**	1553.8 ± 290.9	966.9 ± 485.6	**0.001**
CRP (mg/L)*	2.9 ± 4.5	4.8 ± 5.9	**0.021**
**Glucose (mmol/L)**	5.0 ± 0.6	5.3 ± 0.4	**0.024**
**Insulin (mU/L)**	7.9 ± 2.5	9.4 ± 5.0	0.220

Values are expressed as means ± standard deviation.

*n = 9

**n = 8

***n = 3. When not otherwise indicated values are based on n = 10.
