# Supplementary material for: Thyroid Hormone Activates Brown Adipose Tissue and Increases Non-Shivering Thermogenesis - A Cohort Study in a Group of Thyroid Carcinoma Patients
Source: PLoS One. 2016 Jan 19;11(1):e0145049. doi: 10.1371/journal.pone.0145049 (PMC4718641; doi:10.1371/journal.pone.0145049)
Supplement: S1 Table — (PDF) [file pone.0145049.s002.pdf]

Age_hypo	Age_hyper	BMI_hypo	BMI_hyper	Weight_hypo	Weight_hyper	Bodyfatperc_hypo
50.00	51.00	25.60	25.86	90.60	91.40	26.00
63.00	63.00	35.60	37.41	99.30	103.10	36.50
43.00	44.00	25.80	25.23	73.30	71.20	28.10
34.00	34.00	29.70	29.50	82.70	81.30	34.40
58.00	58.00	33.10	32.04	92.40	89.40	37.40
33.00	33.00	20.30	20.60	56.30	56.80	20.00
59.00	60.00	34.10	34.10	75.70	75.60	44.90
35.00	35.00	21.00	22.10	65.90	69.30	21.20
58.00	58.00	34.10	34.10	80.70	80.70	33.70
41.00	41.00	42.30	40.30	105.60	116.40	55.40

Bodyfatperc_hyper	Fatmass_hypo	Fatmass_hyper	Delta_fatmass	Fatfreemass_hypo
28.00	23608.00	25422.90	1814.90	64461.20
39.90	36396.50	41394.20	4997.70	61198.40
30.30	20786.90	21972.30	1185.40	50707.30
38.20	28829.10	31925.40	3096.30	52467.80
39.30	34629.70	35498.20	868.50	557944.90
20.60	11365.50	11890.90	525.40	43132.40
44.20	34383.10	33660.10	-723.00	404648.40
21.60	14138.40	15053.30	914.90	49959.00
33.80	27508.60	27377.80	-130.80	51904.50
43.20	44995.90	50751.80	5755.90	58927.10

Fatfreemass_hyper	Levothyroxinedose_hypo	Levothyroxinedose_hyper	Interval_Surgery_PETCT__hypo
62531.80	0.00	100.00	7.00
60085.00	0.00	175.00	7.00
47839.90	0.00	150.00	5.00
49192.30	0.00	175.00	5.00
52558.60	0.00	137.50	6.00
43538.10	0.00	150.00	5.00
40976.40	0.00	125.00	7.00
52249.00	0.00	125.00	14.00
51394.60	0.00	137.50	8.00
64265.20	0.00	162.50	4.00

Interval_PETCThypo_PETCThyp	EETN_hypo	EETN_hyper	EECOLD_hypc	EECOLD_hyper	Hypertension_hypc
21.00	4.09	5.22	4.27	6.25	999.00
18.00	3.12	4.32	3.31	5.44	999.00
20.00	3.30	4.25	3.99	5.40	2.00
19.00	4.13	3.89	4.78	4.78	1.00
21.00	4.14	4.17	4.68	5.06	1.00
20.00	3.64	3.90	4.96	5.08	2.00
25.00	2.90	3.65	3.49	4.84	2.00
35.00	4.70	4.45	5.46	5.17	1.00
15.00	3.84	4.83	4.22	5.71	1.00
29.00	3.75	5.32	3.93	6.34	1.00

Hypertension_hyper	RRsystTN_hypo	RRsystTN_hyper	RRsystCOLD_hypo	RRsystCOLD_hypoer
2.00	999.00	136.00	999.00	140.00
2.00	999.00	127.00	999.00	145.00
2.00	106.00	115.00	133.00	106.00
2.00	126.00	107.00	127.00	108.00
1.00	159.00	145.00	159.00	146.00
2.00	115.00	104.00	114.00	113.00
2.00	107.00	113.00	118.00	126.00
1.00	165.00	148.00	183.00	176.00
1.00	148.00	142.00	175.00	142.00
2.00	122.00	118.00	125.00	120.00

RRdiastTN_hypo	RRdiastTN_hyper	RRdiastCOLD_hypo	RRdiastCOLD_hyper	RRsystdelta_hypo
999.00	86.00	999.00	92.00	999.00
999.00	75.00	999.00	75.00	999.00
69.00	68.00	94.00	61.00	27.00
90.00	70.00	95.00	75.00	1.00
119.00	102.00	119.00	105.00	0.00
82.00	68.00	81.00	75.00	-1.00
71.00	67.00	77.00	74.00	11.00
109.00	107.00	110.00	104.00	18.00
109.00	97.00	114.00	101.00	27.00
91.00	77.00	93.00	83.00	3.00

RRsystdelta_hyper	RRdiastdelta_hypo	RRdiastdelta_hyper	MAPTN_hypo	MAPTN_hyper
4.00	999.00	6.00	999.00	103.00
18.00	999.00	0.00	999.00	92.00
-9.00	25.00	-7.00	81.00	84.00
1.00	5.00	5.00	102.00	82.00
1.00	0.00	3.00	132.00	116.00
9.00	-1.00	7.00	93.00	80.00
13.00	0.00	7.00	83.00	82.00
28.00	1.00	-3.00	128.00	123.00
0.00	5.00	4.00	122.00	112.00
2.00	2.00	6.00	101.00	91.00

MAPCOLD_hypo	MAPCOLD_hyper	MAPDelta_hypo	MAPdelta_hyper	HR_TN_hypo	HR_Cold_hypo
999.00	108.00	999.00	5.00	999.00	999.00
999.00	98.00	999.00	6.00	999.00	999.00
107.00	76.00	26.00	-12.00	60.38	61.93
106.00	86.00	4.00	4.00	63.24	61.97
132.00	119.00	0.00	3.00	36.86	36.96
92.00	88.00	-1.00	8.00	999.00	999.00
91.00	91.00	8.00	9.00	37.38	37.43
134.00	128.00	6.00	5.00	36.97	54.44
134.00	115.00	12.00	3.00	72.05	73.18
104.00	95.00	3.00	4.00	999.00	999.00

HR_TN_hyper	HR_Cold_hyper	HR_delta_hypo	HR_delta_hyper	TmeanskinTN_hypo
999.00	999.00	999.00	999.00	32.15
999.00	999.00	999.00	999.00	31.60
999.00	999.00	1.55	999.00	32.26
67.94	67.78	-1.27	-0.16	31.91
999.00	999.00	0.10	999.00	32.39
999.00	999.00	999.00	999.00	32.73
999.00	999.00	0.05	999.00	31.98
58.65	54.86	17.46	-3.89	32.19
73.63	75.71	1.13	2.08	32.50
75.00	69.78	999.00	-8.16	32.61

TmeanskinCOLD_hypo	TmeanskinTN_hyper	TmeanskinCOLD_hyper	TCoreTN_hypo	TCoreTN_hyper
29.86	33.73	30.29	37.03	999.00
28.88	33.19	31.55	36.51	36.33
28.53	33.33	30.41	36.99	37.18
29.51	32.51	30.25	37.71	36.99
30.19	33.29	30.26	36.88	36.93
29.90	33.78	30.52	999.00	37.08
29.06	32.60	30.48	37.44	36.70
29.85	33.42	31.01	36.98	37.08
30.35	33.33	31.79	37.79	37.40
29.23	33.38	32.17	36.90	40.53

TCoreCOLD_hypo	TCoreCOLD_hyper	TCoreTN_delta	TCoreCOLD_delta	TCoreminskinTN_hypo
37.52	999.00	0.50	999.00	5.07
36.56	36.11	0.06	-0.22	4.82
999.00	37.63	999.00	0.45	999.00
37.96	37.96	0.25	0.97	5.80
37.16	37.13	0.29	0.21	4.48
999.00	37.08	999.00	0.00	999.00
37.41	36.79	-0.03	0.08	2.99
37.30	37.66	0.32	0.57	4.78
39.01	37.71	1.23	0.31	5.27
36.82	41.04	-0.16	0.51	4.30

TCoreminskinTN_hyper	TCoreminskinCOLD_hypo	TCoreminskinCOLD_hyper	Tcoreminskindelta_hypo
999.00	7.66	999.00	2.59
3.14	7.68	999.00	2.86
2.95	999.00	999.00	999.00
4.49	8.42	7.00	2.62
3.61	6.76	6.91	2.29
3.29	999.00	6.55	999.00
5.25	8.37	6.35	1.75
3.56	7.33	6.48	2.55
5.24	8.67	5.85	-3.26
6.86	7.59	9.80	2.64

Tcoreminskindelta_hyper	RelativeSPhandTN_hypo	RelativeSPfhandTN_hyper	RelativeSPhandCOLD_hypo
999.00	1.00	1.00	0.33
999.00	1.00	1.00	0.53
999.00	1.00	1.00	0.44
2.50	1.00	1.00	0.20
3.30	1.00	1.00	0.57
3.24	1.00	1.00	0.19
6.00	1.00	1.00	0.04
2.93	1.00	1.00	0.43
2.99	1.00	1.00	0.48
2.96	1.00	1.00	0.90

RelativeSPhandCOLD_hyper	RelativeSPhanddelta_hypo	RelativeSPhanddelta_hyper
0.08	-0.67	-0.92
0.26	-0.47	-0.74
0.11	-0.56	-0.89
0.19	-0.80	-0.81
0.30	-0.43	-0.70
1.98	-0.81	0.98
999.00	-0.96	999.00
1.39	-0.57	0.39
0.60	-0.52	-0.40
0.70	-0.10	-0.30

BAT_SUVmean_hypo	BAT_SUVmean_hyper	BAT_SUVmeandelta	FFA_hypo	FFA_hyper	FFAdelta
1.01	1.77	0.76	681.43	598.81	82.62
1.58	1.81	0.23	510.05	476.77	33.28
2.60	3.69	1.09	633.86	596.43	37.43
6.99	8.41	1.42	534.78	527.12	7.66
1.31	1.43	0.12	605.66	907.25	-301.59
3.38	8.24	4.86	821.66	524.04	297.62
2.58	2.66	0.08	932.97	372.28	560.69
1.56	1.77	0.21	915.95	282.98	632.97
1.20	2.50	1.30	952.36	659.79	292.57
1.30	7.47	6.17	750.16	673.00	77.16

TG_hypo	TG_hyper	TGdelta	CRP_hypo	CRP_hyper	Glucose_hypo	Glucose_hyper	Insulin_hypo
2096.98	1251.80	845.18	0.43	1.25	4.69	5.23	7.18
1356.46	716.92	639.80	0.10	0.60	5.57	5.62	9.21
1632.85	761.05	871.80	0.12	0.40	4.30	5.28	3.47
1365.46	73.00	1292.46	0.12	0.60	4.69	5.08	9.31
1087.78	904.72	183.06	0.50	3.28	5.11	5.35	6.55
1426.10	452.82	973.28	0.30	0.91	4.43	5.18	5.74
1554.28	1563.60	-9.32	2.70	4.62	5.72	5.49	8.38
1905.70	1622.19	283.51	14.03	16.00	5.94	5.67	6.91
1437.20	1097.91	339.29	3.16	5.66	4.20	4.64	11.83
1675.64	1224.90	450.74	7.28	15.01	5.37	5.95	10.72

Insulin_hyper	NoradrenalinTN_hypo	NoradrenalinCOLD_hypo	Noradrenalindelta_hypo
10.57	14.00	4.90	-9.40
9.18	5.07	9.32	4.25
5.68	6.24	11.40	5.16
4.42	999.00	7.66	999.00
7.44	3.67	8.38	4.71
5.18	1.85	4.17	2.32
9.57	4.42	5.53	1.11
6.55	2.23	5.36	3.13
20.56	3.20	3.33	0.13
15.16	1.40	2.31	0.91

NoradrenalinTN_hyper	NoradrenalinCOLD_hyper	Noradrenalindelta_hyper	AdrenalinTN_hypo
2.48	4.26	1.78	0.10
3.58	5.06	1.48	0.32
4.67	7.15	2.48	0.14
1.43	2.34	0.91	999.00
1.37	3.77	2.40	0.16
1.00	2.19	1.19	0.34
2.00	1.85	-0.15	0.19
2.05	3.63	1.58	0.30
1.78	999.00	999.00	0.20
1.03	2.11	1.08	0.15

AdrenalinCOLD_hypo	Adrenalindelta_hypo	AdrenalinTN_hyper	AdrenalinCOLD_hyper
0.49	0.39	0.33	0.19
0.17	-0.15	0.01	0.07
0.11	-0.03	0.10	0.06
0.28	999.00	0.08	0.08
0.12	-0.04	0.10	0.06
0.25	-0.09	0.14	0.14
0.09	-0.10	0.07	0.05
0.24	-0.06	0.14	0.17
0.18	-0.02	0.15	999.00
0.09	-0.06	0.08	0.06

Adrenalindelta_hyper	TSH_hypo	TSH_hyper	TSHdelta	fT4_hypo	fT4_hyper	fT4delta
-0.14	124.20	0.00	124.20	3.00	25.00	22.00
0.06	999.00	999.00	999.00	25.00	25.80	0.80
-0.04	117.40	1.70	115.70	2.50	23.00	20.50
0.00	190.00	0.10	189.90	3.00	26.50	23.50
-0.04	52.60	0.00	52.60	3.40	27.20	23.80
0.00	107.00	0.00	107.00	3.70	24.80	21.10
-0.02	160.60	0.80	159.80	3.30	21.60	18.30
0.03	13.40	0.10	13.30	5.30	23.20	17.90
999.00	140.60	1.00	139.60	3.40	19.50	16.10
-0.02	62.60	0.80	61.80	3.50	14.20	10.70
