# Supplementary material for: Thyroid Hormone Activates Brown Adipose Tissue and Increases Non-Shivering Thermogenesis - A Cohort Study in a Group of Thyroid Carcinoma Patients
Source: PLoS One. 2016 Jan 19;11(1):e0145049. doi: 10.1371/journal.pone.0145049 (PMC4718641; doi:10.1371/journal.pone.0145049)
Supplement: S1 Text — (PDF) [file pone.0145049.s003.pdf]

C1

Brown adipose tissue activity and thyroid hormone

(Bruin vet activiteit en schildklierhormoon)

Version 4

29 February 2012

Maastricht University

**Short title**	Brown adipose tissue activity and thyroid hormone
**Version**	4
**Date**	February 2012
**Project leader**	W.D. van Marken Lichtenbelt, PhD, Dept of Human Biology, MUMC+
**Principal investigator**	W.D. van Marken Lichtenbelt, PhD, Dept of Human Biology, MUMC+
**Independent physician**	S. Landewé-Cleuren
**Laboratory sites**	Dept of Human Biology, Maastricht University<br>Dept of Nuclear Medicine, Maastricht University Medical Centre
**Sponsor**	Maastricht University
**Project team**	• W.D. van Marken Lichtenbelt, PhD, Dept of Human Biology, MUMC+<br>E-mail: markenlichtenbelt@maastrichtuniversity.nl<br>• B. Havekes, MD, PhD, Dept of Endocrinology, MUMC+<br>E-mail: bas.havekes@mumc.nl<br>• Prof. N. Schaper, MD, PhD, Dept of Endocrinology, MUMC+<br>E-mail: n.schaper@mumc.nl<br>• M. Kars, MD, PhD, Dept of Endocrinology, MUMC+<br>E-mail: m.kars@mumc.nl<br>• N.D. Bouvy, MD, PhD, Dept of Surgery, MUMC+<br>E-mail: n.bouvy@mumc.nl<br>• B. Brans, MD, PhD, Dept of Nuclear Medicine, MUMC+<br>E-mail: b.brans@mumc.nl<br>• Prof. P. Schrauwen. PhD, Dept of Human Biology, MUMC+<br>E-mail: p.schrauwen@maastrichtuniversity.nl<br>• G.H.E.J. Vijgen, MD, Dept of Surgery / Dept of Human Biology, MUMC+<br>E-mail: g.vijgen@maastrichtuniversity.nl

PROTOCOL SIGNATURE SHEET

Name	Signature	Date
**Principal Investigator:**<br><br>W.D. van Marken Lichtenbelt, PhD<br>Dept of Human Biology<br>Maastricht University<br>Universiteitssingel 50, k. 2.350<br>PO Box 616<br>6200 MD<br>Maastricht<br>Tel +31 43 3881629<br>E-mail:<br>markenlichtenbelt@maastrichtuniversity.nl		

TABLE OF CONTENTS

1. SUMMARY 1
2. INTRODUCTION AND RATIONALE 2
3. OBJECTIVES 6
   3.1 Primary Objective: 6
   3.2 Secondary Objective(s): 6
4. STUDY DESIGN 6
5. STUDY POPULATION 10
   5.1 Population 10
   5.2 Inclusion criteria 10
   5.3 Exclusion criteria 10
   5.4 Sample size calculation 10
6. METHODS 11
   6.1 Study parameters/endpoints 11
      6.1.1 Main study parameter/endpoint 11
      6.1.2 Secondary study parameters/endpoints 11
      6.1.3 Other study parameters 11
   6.2 Study procedures 11
   6.3 Measurements 12
      6.3.1 Respiration chamber & energy expenditure 12
      6.3.2 Body temperature distribution 12
      6.3.3 Heart rate 13
      6.3.4 FDG PET-CT scan 13
      6.3.5 Body composition 13
      6.3.6 Blood sample 13
   6.4 Withdrawal of individual subjects 13
7. RISKS OF STUDY PROTOCOL 14
   7.1 Risks of FDG-PET-CT and DXA scan 14
8. SAFETY REPORTING 14
   8.1 Section 10 WMO event 14
   8.2 Adverse and serious adverse events 14
   8.3 Follow-up of adverse events 15
9. STATISTICAL ANALYSIS 16
   9.1 Descriptive statistics 16
   9.2 Univariate analysis 16
   9.3 Multivariate analysis 16
10. ETHICAL CONSIDERATIONS 17
   10.1 Regulation statement 17
   10.2 Recruitment and consent 17
   10.3 Compensation for injury 18
11. ADMINISTRATIVE ASPECTS AND PUBLICATION 19
   11.1 Handling and storage of data and documents 19
   11.2 Amendments 19

11.3 Annual progress report 19
11.4 End of study report 19
11.5 Public disclosure and publication policy 20
12. REFERENCES 21

LIST OF ABBREVIATIONS AND RELEVANT DEFINITIONS

ABR	ABR form, General Assessment and Registration form, is the application form that is required for submission to the accredited Ethics Committee (In Dutch, ABR = Algemene Beoordeling en Registratie)
AE	Adverse Event
AR	Adverse Reaction
BAT	Brown Adipose Tissue
BMI	Body Mass Index
CCMO	Central Committee on Research Involving Human Subjects; in Dutch: Centrale Commissie Mensgebonden Onderzoek
CV	Curriculum Vitae
DXA	Dual-Energy-X-ray-Absorptiometry
^{18}F-FDG	2-deoxy-2-^{18}F-fluoro-D-glucose, a radioactive labeled glucose tracer, is used in nuclear imaging (PET-CT) to depict glucose uptake for tumor diagnosis
FT4	free T4, thyroxine, the parent form of thyroid hormone, converted into the active hormone, T3
kBq	Kilo Bequerel, unit for radiologic disintegration
EU	European Union
GCP	Good Clinical Practice
IC	Informed Consent
I^{124}	A proton-rich isotope of iodine used as a radiochemical for determination of thyroid gland remnants after thyroid gland resection for well-differentiated thyroid gland carcinoma.
I^{131}	Radioactive iodine, used for radioactive ablation therapy of thyroid gland remnants after thyroid gland resection for well-differentiated thyroid gland carcinoma
METC	Medical research ethics committee (MREC); in Dutch: medisch ethische toetsing commissie (METC)
PET-CT	Positron-emission-tomography and computed-tomography.
(S)AE	(Serious) Adverse Event
SUSAR	Suspected Unexpected Serious Adverse Reaction SUV
SUV	Standard Uptake Value, outcome parameter of PET-CT-imaging. Defined as calculated by uptake (kBq/mL) / injected dose (kBq) / patient weight (g))
T3	Triiodothyronine, active form of thyroid hormone
TSH	Thyroid-stimulating hormone
WBP	Personal Data Protection Act (in Dutch: Wet Bescherming Persoonsgevens)
WMO	Medical Research Involving Human Subjects Act (in Dutch: Wet Medisch-wetenschappelijk Onderzoek met Mensen

1. SUMMARY

Rationale: During the last decades, research in possible therapies for existing obesity and developmental factors causing obesity has explosively increased. Recently renewed interest aroused for a tissue playing a possible role in both development and therapy for obesity: brown adipose tissue (BAT).

To define the relation between BAT and thyroid hormone, we set up the following research protocol. In this protocol BAT activity will be determined in subjects that underwent thyroidectomy for well-differentiated thyroid carcinoma.

Objective: To study the effect of thyroid hormone and thyroid-stimulating hormone on brown adipose tissue activity.

Study design: Determine BAT activity after thyroidectomy in well-differentiated thyroid carcinoma patients.

Study population: Patients that underwent thyroidectomy for well-differentiated thyroid carcinoma, male and female, aged 18-65 years.

Intervention: FDG-PET-CT-imaging of BAT activity will be performed under cold stimulation twice.

For patients clinically withdrawn from thyroid hormone suppletion, the first occasion will be in a hypothyroid state within 4-6 weeks after thyroidectomy and the second measurement will take place in a euthyroid state 4 months after the start of thyroid hormone treatment.

For patients receiving recombinant-thyroid-stimulating-hormone injections, the first occasion will be shortly after the injection in a state of high thyroid-stimulating hormone levels. The second measurement will be in a euthyroid state 4 months after the injection.

Main study parameters/endpoints: The main endpoint of this study is the effect of thyroid hormone and thyroid-stimulating hormone on BAT activity in kBq and SUV. Secondary endpoints are the effects of thyroid hormone and thyroid-stimulating hormone on energy metabolism, body core temperature, skin surface temperature and skin perfusion.

Nature and extent of the burden and risks associated with participation, benefit and group relatedness: The absorbed radiation dose from the FDG PET-CT scan after administration of 74 MBq of ^{18}F-FDG is 2.8 mSv. The routine clinical treatment program requires a I^{124} PET-CT scan (182mSv) and a I^{131} ablation therapy (41625 mSv) of possible thyroid gland remnants. The additional radiation dose from measurements in this study is considered low in comparison to the standard radiation dose given for regular therapy.

2. INTRODUCTION AND RATIONALE

Obesity is number two preventable cause of death in Western society. Therefore, the treatment of this chronic disease and its comorbidities has high priority. The essence of obesity consists of an imbalance between the ingestion and combustion of food. Hence, the quest for new therapeutic targets to correct this disequilibrium receives strong scientific attention. On the one hand, a high number of therapies focus on decreasing energy intake (ingestion), but on the other hand increasing energy expenditure (combustion) could also be a valuable contributor.

Brown adipose tissue (BAT) is suggested to be an important regulator of the energy balance. In response to cold exposure, BAT is able to produce heat to prevent hypothermia. In response to food, BAT is suggested to produce heat to prevent obesity. After infancy the amount of BAT decreases and it was always believed to gradually become dysfunctional. Strikingly, BAT has recently shown to be present in significant amounts and becomes functionally active in adult man upon cold exposure.[1-3] 2-deoxy-2-^{18}F-fluoro-D-glucose (^{18}F-FDG), a radioactive labeled glucose tracer, is used in nuclear imaging (positron-emission-tomography-and-computed-tomography (PET-CT)) to depict glucose uptake for tumor diagnosis. Frequently non-malignant uptake in adipose tissue was seen, which later appeared to be related to mild cold conditions during the tracer administration. Tissue biopsies from PET-active supraclavicular regions confirmed BAT presence, responsible for the glucose uptake.[2,3] Since this discovery the interest in the possibilities of this thermogenic organ increased exponentially. Because of its high heat production capacity in rodents,[4] the stimulation of BAT in man is seen as an anti-obesity target.[5,6]

Interestingly, the amount of cold induced BAT activity in man shows large individual variation. Of special interest is the strong negative correlation with body fat percentage, suggesting the involvement of BAT in obesity.[2,7] Here, low BAT activity could be associated with low total daily energy expenditure. If low BAT activity is responsible for this weight increase, correcting BAT dysfunction could be an important prevention target for obesity.

In addition to direct stimulation by sympathetic innervation, endocrine stimulation could also considerably contribute to BAT thermogenesis. The thyroid gland secretes thyroid hormone, which increases energy expenditure in man. The effect of thyroid hormone on energy expenditure in man is significant, as illustrated in clinical states of hypo- or hyperthyroidism where energy expenditure can decrease or increase up to three times compared to baseline.[8] The thyroid gland mainly secretes the inactive pro-hormone thyroxine (T4), that in the target cells needs to be deiodinised (lose an iodine atom) by deiodinases to the active hormone T3. Treatment of human stem cells with T3 stimulates the development of UCP-1-positive cells in white adipose tissue.[9] Since UCP-1 is the protein that facilitates

mitochondrial uncoupling in BAT, this suggests thyroid hormone could significantly affect BAT functionality in man.

In rodents, thyroid hormone can stimulate BAT. After conversion of T4 to T3 by the BAT-specific type-II-iodothyronine-5-deiodinase (D2), thyroid hormone enhances BAT thermogenesis.[10,11] Thyroid-stimulating hormone (TSH), secreted by thyrotrope cells in the anterior pituitary gland, stimulates the thyroid gland to release T4. Interestingly, TSH itself is also involved in thermogenesis and TSH-receptors are present on BAT in rats.[12] This suggests both TSH and T4 might be important in BAT function.

Nevertheless, without thyroid stimulation BAT can still become active. If D2-deficient mice are exposed to cold, thermogenesis is still present, stimulated by the sympathetic stimulation. However, if cold exposure is absent glucose intolerance, non-alcoholic fatty liver disease (NAFLD) and diet-induced obesity develop.[13] This suggests that thyroid-induced, D2-dependent BAT activity is important to prevent development of these diseases.

Besides the direct (systemic) influence of thyroid hormone on the BAT cell, thyroid hormone could also be a central inducer of BAT by directly stimulating the hypothalamic pathway. This process especially seems to be important under thermoneutral conditions when there is no cold stimulus.[14] Therefore, thyroid function seems to work on BAT activity both at cellular level during cold exposure and through the brain regardless of temperature. Support for this hypothesis is given by the case report of a patient with extreme insulin resistance and thyroid cancer. The therapeutic treatment with high doses of thyroid hormone was accompanied with active BAT on PET-CT, without cold exposure.[15] Interestingly, thyroid hormone treatment also resolved the hyperglycaemia of this patient, suggesting a role for BAT in glucose metabolism. In a larger study cohort, increased insulin resistance was associated with a D2-gene mutation. Moreover, if this D2-mutation was accompanied by a mutation in the ß-3-adrenergic receptor, body mass index (BMI) was significantly larger.[16]

In summary, the interplay between the sympathetic nervous system and thyroid function is important in the control of BAT activity. During hypothyroidism, BAT activity could be impaired, which could lead to overweight and associated comorbidities (e.g. insulin resistance). If safe, BAT-targeted thyroid thermogenesis could increase energy expenditure.[17] However, besides the single case report of Skarulis et al. (see above), no prospective studies have confirmed the strong relation between thyroid function and BAT activity, as was observed in rodents, in man yet. Therefore, we hypothesize that thyroid hormone is related to the level of BAT activity in man.

Interestingly, large changes in thyroid hormone levels are routinely observed in patients treated with a total thyroidectomy and subsequent radioactive iodine ablation for well-differentiated thyroid carcinoma.

Part of the regular, clinical postoperative program is the ablation treatment with ^{131}iodine to eliminate possible thyroid gland tissue remnants. Based on the individual case, the patient's endocrinologist and surgeon decide to follow one of the two available treatment protocols for ^{131}iodine ablation.

1. The first option comprises withdrawal from thyroid hormone for 4-6 weeks in order to improve effectiveness of adjunctive radioiodine ablation by stimulating the endogenous TSH response. In short, every patient will develop a hypothyroid state after complete resection of the thyroid gland in order to be able to effectively eradicate possible thyroid remnants with radioactive iodine ablation therapy. Therefore, after a total thyroidectomy for well-differentiated thyroid carcinoma thyroid hormone therapy is not started immediately after surgery. Subsequently, thyroid-stimulating hormone (TSH) will drastically increase in combination with a decrease in free thyroxine (FT4) in the first weeks post-resection. The high levels of TSH will then maximally stimulate the thyroid remnant to incorporate iodine and thus increase the effect of the administered radioactive iodine dose. Approximately 4-6 weeks post-resection, radioactive iodine (I^{131}) ablation therapy is performed to inactivate all (microscopic) thyroid gland remnants. After administering the I^{131}-therapy, thyroid hormone suppletion with levothyroxine (Euthyrox®) is started in TSH-suppressive doses to normalize thyroid hormone levels to the high-normal range and decrease plasma TSH to levels <0.1 mU/L. This TSH-suppression is done to prevent TSH-mediated growth of (possibly) persistent thyroid gland tissue. In most cases, patients are free of tumor after these therapies and have an excellent prognosis. After ablation, TSH-suppressive doses of thyroid hormone are instituted to reduce the potentially detrimental effects of TSH on remnant thyroid cells (if present after therapy).

   We hypothesize that BAT activity decreases after thyroid hormone withdrawal and increases upon thyroid hormone suppletion.

2. The second option comprises direct postoperative institution of thyroid hormone without a period of thyroid hormone withdrawal. To induce high levels of plasma TSH, injections with recombinant-TSH are given before the radioactive iodine ablation (electively planned). The high levels of TSH (in the presence of normal to high-normal thyroid hormone levels) will then maximally stimulate the thyroid remnant to incorporate iodine and thus increase the effect of the administered radioactive iodine dose,. One to two days after the injections the ^{131}iodine ablation treatment for possible elimination of thyroid gland tissue remnants is given. Suppletion with levothyroxine (Euthyrox®) is administered in TSH-suppressive doses to normalize thyroid hormone levels to the high-normal range and decrease plasma TSH to levels <0.1 mU/L.

   We hypothesize that BAT activity increases upon recombinant-TSH injections.

We think these cohorts are very interesting and offer a unique opportunity to observe the effect of thyroid hormone and/or TSH on BAT activity. Also, observing the effect of TSH-injections will allow for determination of the specific effect of TSH on BAT activity. This study will observe well-differentiated thyroid carcinoma patients that are selected for total thyroidectomy according to national and local oncology guidelines as defined in the Dutch oncology guidelines for thyroid carcinoma (http://www.oncoline.nl/schildkliercarcinoom). In addition to these national guidelines, the Maastricht University Medical Centre (MUMC+) routinely performs a ^{124}I-scintigraphy to visualize possible thyroid remnants and/ or distant metastases, all as part of the regular follow-up after total thyroidectomy.

3. OBJECTIVES

3.1 Primary Objective:

To study the effect of thyroid hormone on brown adipose tissue activity.

3.2 Secondary Objective(s):

To study the effect of thyroid hormone on energy metabolism, body core temperature, skin surface temperature and skin perfusion.

4. STUDY DESIGN

In this study BAT activity is analyzed during the two different phases where thyroid hormone and/or TSH is either low or normal-to-high after total thyroidectomy. Depending on the treatment chosen by the patient and the consulting endocrinologist and surgeon, two protocols can be followed (1. Withdrawal of thyroid hormone and 2. Recombinant TSH-injection, see *Introduction).* We will observe BAT activity in both treatment groups (1 and 2), as follows:

Protocol 1. Withdrawal (Figure 1):

1. First determination of BAT activity in the hypothyroid state 4-6 weeks after resection,
2. Second determination of BAT activity in the euthyroid near-to-hyperthyroid with TSH <0.1 mU/L 4 months after ^{131}I-therapy.

This will allow us to determine the changes on BAT activity induced by changes in thyroid hormone level.

However, this protocol 1. does not completely exclude a possible effect of the markedly raised TSH *per se* that develops after thyroidectomy. Therefore, we will also observe those subjects that are not withdrawn from thyroid hormone suppletion, but receive recombinant-TSH injections without thyroid hormone withdrawal. This will allow us to determine the effects of TSH on BAT activity (see also *Protocol 2* below).

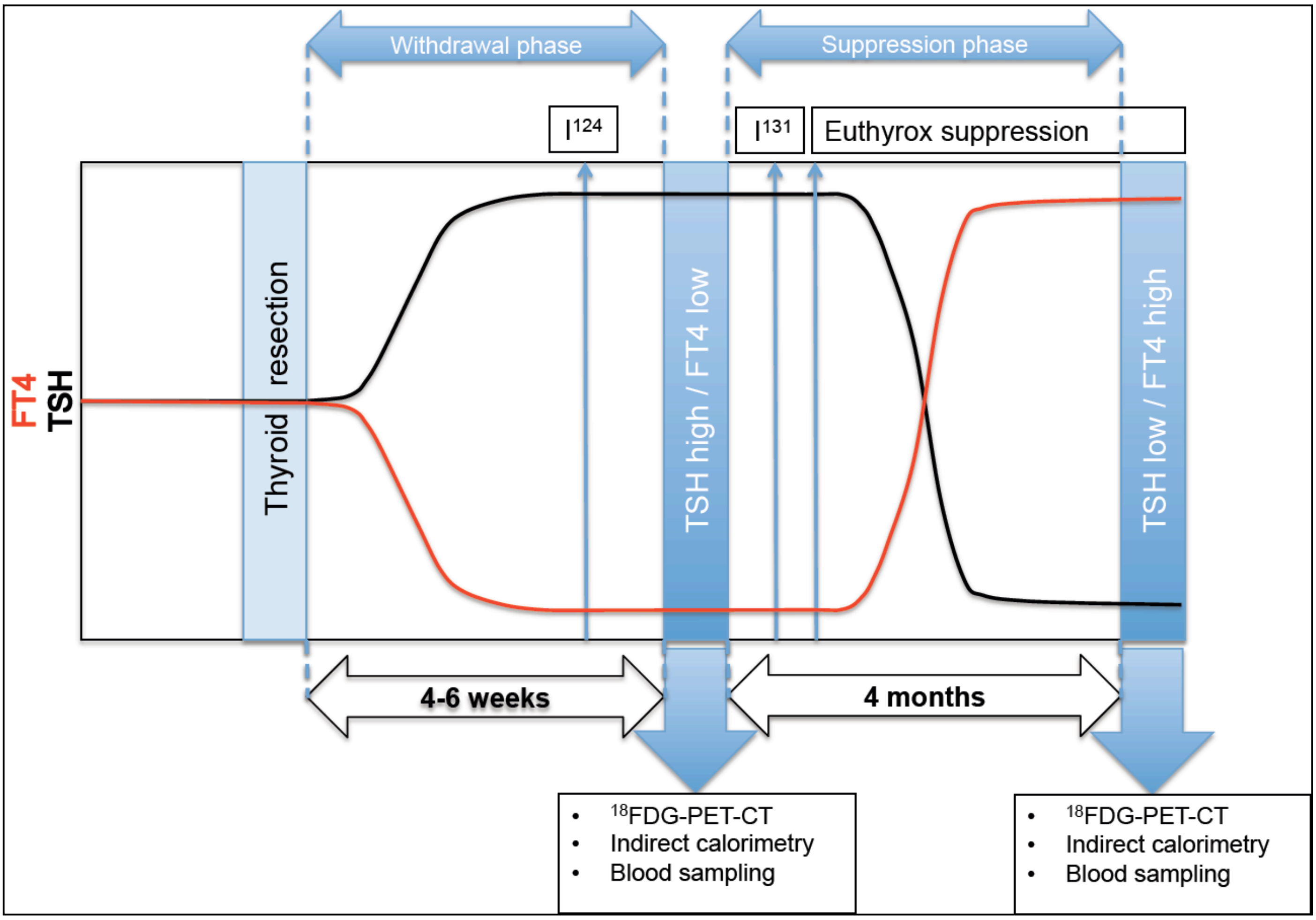

Figure 1. Schematic representation of study measurements after total thyroidectomy in the Maastricht University Medical Centre. Blue arrows indicate moment of study measurements. FT4 indicates free thyroxine, TSH indicates thyroid-stimulating hormone, Euthyrox® indicates pharmacological levothyroxine suppletion that suppresses endogenous TSH. I^{131} indicates radioactive iodine, used for radioactive ablation therapy of thyroid gland remnants after thyroid gland resection for well-differentiated thyroid gland carcinoma. I^{124} indicates a proton-rich isotope of iodine used as a radiochemical for determination of of thyroid gland remnants after thyroid gland resection for well-differentiated thyroid gland carcinoma.

Protocol 2. Recombinant TSH-injection (Figure 2):

3. First determination of BAT activity after recombinant-TSH-injections (electively planned after thyroid gland resection).
4. Second determination of BAT activity in the euthyroid near-to-hyperthyroid with TSH <0.1 mU/L 4 months after ^{131}I-therapy.

This will allow us to determine which effect TSH levels specifically have on BAT activity.

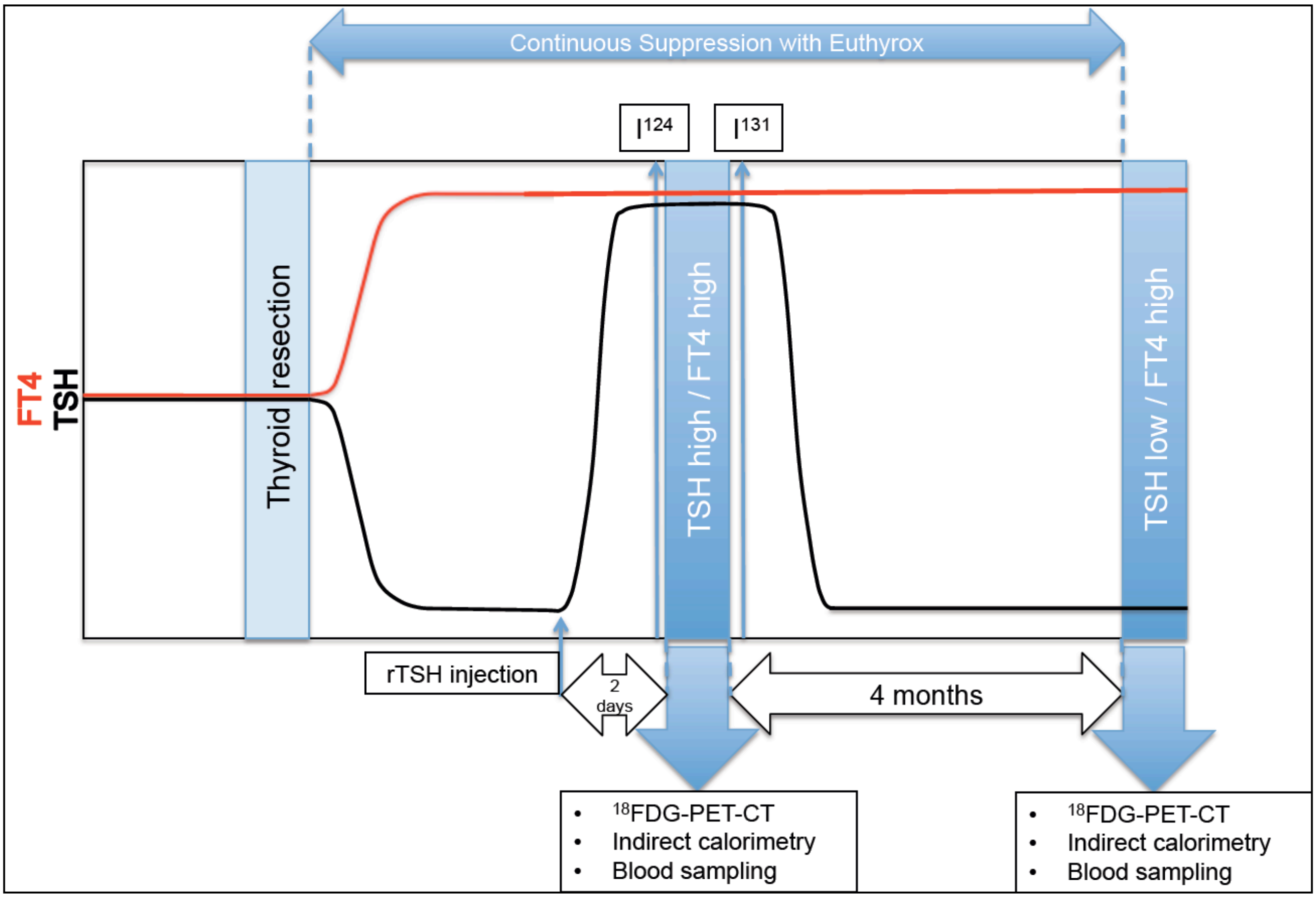

Figure 2. Schematic representation of study measurements after total thyroidectomy in the Maastricht University Medical Centre. Blue arrows indicate moment of study measurements. FT4 indicates free thyroxine, TSH indicates thyroid-stimulating hormone, Euthyrox® indicates pharmacological levothyroxine suppletion that suppresses endogenous TSH. rTSH indicates recombinant-TSH injected several days before I^{131}-ablation therapy. I^{131} indicates radioactive iodine, used for radioactive ablation therapy of thyroid gland remnants after thyroid gland resection for well-differentiated thyroid gland carcinoma. I^{124} indicates a proton-rich isotope of iodine used as a radiochemical for determination of thyroid gland remnants after thyroid gland resection for well-differentiated thyroid gland carcinoma.

The study measurements will be performed in a cohort of patients undergoing elective total thyroidectomy as indicated for well-differentiated thyroid carcinoma, according to the regular clinical follow-up as described in established guidelines (see *Introduction*). The MUMC+ has extensive experience in the treatment of these patients in the last decades. Currently, approximately 5-10 total thyroidectomies for well-differentiated thyroid carcinoma are performed each year. In addition, other thyroid gland surgery is routinely performed in the MUMC+ as well, approximately 50 thyroid gland operations are performed every year.

All patients undergoing total thyroidectomy are seen and followed-up in the endocrinology department by Dr. B. Havekes or Dr. M. Kars and are treated by the surgeon Dr. N.D. Bouvy. When the patients are informed about their diagnosis in the outpatient department and are indicated for total thyroidectomy for well-differentiated thyroid carcinoma, one of the before mentioned physicians (Dr. B. Havekes, Dr. M. Kars) will inform the patient about this study. If the patient is interested, the subject information letter is handed to the patient in the outpatient clinic. After a time window of two weeks the patient will be informed by telephone by the researcher (G. Vijgen) about his/her participation. If the patient is willing to participate, the informed consent form can be signed and sent to the researcher by mail. The informed consent form is then stored and the patient is included in the study.

There can be a time window between the visit in the outpatient clinic and the actual moment of surgery and subsequent withdrawal/rTSH therapy. Therefore, after inclusion as described above the subject will be re-contacted by telephone by the researcher two weeks before the study measurements are performed (2-4 weeks after surgery). At this moment, the subject will be asked to re-confirm informed consent to be sure the subject still wants to participate in the study. In addition, the physician (Dr. B. Havekes or Dr. M. Kars) will need to confirm that the subject is in a stable medical and psychological condition to be able to participate in the study. If the subject does not want to participate or is in an unstable condition (as judged by the treating specialist), the contact with the researcher ends. If the patient wants to participate, both measurements are planned by the researcher in accordance with the clinical treatment program.

Eighteen patients undergoing total thyroidectomy for well-differentiated thyroid carcinoma will be asked for two observational measurements. Both measurements will be conducted at the department of Nuclear Medicine of the MUMC+.

5. STUDY POPULATION

5.1 Population

18 Patients undergoing a total thyroidectomy for thyroid gland cancer.

5.2 Inclusion criteria

- Male or postmenopausal females undergoing a total thyroidectomy for well-differentiated thyroid carcinoma
- Age 18-65 years
- Stable physical activity levels for at least six months
- **Note:** In case of use of anticoagulation, the dose will be adjusted according to plasma thyroid hormone values.

5.3 Exclusion criteria

- Psychologically unstable subjects (as judged by the treating medical specialist)
- Subjects with mental retardation (as judged by the treating medical specialist)
- Subjects with severe behavior disorders (as judged by the treating medical specialist)
- Pregnant subjects
- The use of the following medication is an exclusion criterium;
    - ß-blockers
- Participation in an intensive weight-loss program or vigorous exercise program during the last year before the start of the study
- Abuse of drugs and/or alcohol
- Severe diabetes which requires application of insulin or patients with diabetes-related complications

5.4 Sample size calculation

At this moment there is only one report on data regarding the effect of thyroid function on BAT activity in man. Therefore, we will use the data from our previous prospective studies to make an estimation of the required sample size. A previous study showed that people respond differently to cold. BAT activity versus no BAT activity was 228 kBq with a standard deviation (SD) of 304 kBq. In the present study, the same difference is expected upon high or low levels of TSH or T4. With this information, an alpha of 0.05 and a power of 0.8, the group of patients should consist of 13 people. Regarding possible dropouts a total number of 18 people will be included.

6. METHODS

6.1 Study parameters/endpoints

6.1.1 Main study parameter/endpoint

The main endpoint of this study is BAT activity expressed in Standard Uptake Value (SUV) after total thyroidectomy.

6.1.2 Secondary study parameters/endpoints

Secondary endpoints are energy metabolism, body composition (weight, body fat), body core temperature, skin surface temperature and skin perfusion.

6.1.3 Other study parameters

Other relevant study parameters are medical history, medication, current medical status, age, weight history, height, sex and thyroid gland function.

6.2 Study procedures

After informed consent is obtained, subjects are invited for the study protocol twice (see also Study Design). Measurements will be planned in accordance with the clinical treatment protocol.

Study protocol

The volunteers will be studied in a fasting state in the morning after an overnight fast. Subjects are instructed to wear light clothing that covers their arms and legs. The subjects come in the study room located in the department of Nuclear Medicine. First, a DXA-scan will be made to determine body composition (Table 1). This scan requires a low dose of x-ray radiation.

Next, iButtons (type DS1921H; Maxim, TX, USA) are attached to measure skin temperatures continuously at the 14 ISO-defined positions[18]. Subjects ingest a telemetric pill that measures core temperature in the intestines via a wireless connected measurement system (Coretemp, HQ Inc, FL, USA). Heart rate will also be monitored with the same system via a chest band.

Next, for later attenuation of the FDG-PET-CT-scan a catheter is injected in the antecubital vein of one of the upper limbs. When the catheter is placed, 10mL of blood is withdrawn for analysis of fasting glucose and thyroid gland function (TSH and T4).

Next, subjects enter the respiration chamber where they are seated in a supine position in a nephrodialysis chair. During measurements, subjects are allowed to watch television. In the following three hours, energy expenditure will be measured with a respiratory gas analyzer using a ventilated hood system (Oxycon, Jaeger, Germany). The first 60 minutes are measured under thermoneutral conditions room temperature of 24ºC). After the first hour of measurements under thermoneutral conditions, air temperature is decreased until the subject starts shivering. In previous studies the average time until shivering was approximately 25 minutes.[7] After the onset of shivering, temperatures are stabilized just above shivering level to induce maximal non-shivering thermogenesis. After the second hour circa 74 MBq (2 mCi) ^{18}F-FDG is injected via the catheter. After injection, subjects are instructed to lay still to prevent muscle uptake of ^{18}F-FDG. After another hour of measurements under mild cold conditions (one hour after the application of the FDG) the PET-CT-scan will be made. The scanning protocol starts with a low dose CT-scan, immediately followed by a PET scan (6-7 bed positions, each lasting 6 minutes). Total scan time thus will last 36 to 42 minutes, depending on the size of the volunteer. The CT scan will be used for attenuation correction and localization of the ^{18}F-FDG uptake sites.

Table 1. Measurements performed during both analyses in this study.

	1st analysis	2nd analysis
DXA-scan	X	X
FDG-PET-CT-scan	X	X
Energy expenditure	X	X
Blood sample	X	X

6.3 Measurements

6.3.1 Respiration chamber & energy expenditure

Measurements will take place in a transportable climate chamber under thermoneutral conditions and during mild cold exposure.[19] Energy expenditure will be determined using a ventilated hood system (Oxycon, Jaeger, Germany).

6.3.2 Body temperature distribution

Core temperature is assessed by means of temperature telemetry using commercially available medical grade capsules (CorTemp™). Skin temperature is measured by wireless iButton data loggers (iButton®)[20]. Mean skin temperature is

calculated using skin temperatures measured at the 14 sites prescribed by the ISO-standard.[21]

6.3.3 Heart rate

To assess possible heart rate variation this is recorded with a heart rate monitoring belt, that is connected and registered by the CorTemp system.

6.3.4 FDG PET-CT scan

^{18}F FDG PET-CT scans will be made using an advanced Gemini CT-PET scanner (Philips, Cleveland). Imaging will be performed in 3D mode, with emission scans of 6 minutes per bed position and 6-7 bed positions are needed to cover the areas where brown fat tissue is usually found (neck, thorax, and around the kidneys).

6.3.5 Body composition

Body composition will be measured by DXA scan (Dual Energy X-ray Absorptiometry) at the department of Nuclear Medicine [22]. This a non-invasive technique. The subject lay in supine position for approximately 10 minutes. The effective radiator dose is, depending on the duration of the scan (size of the body) between 1-7 microSievert (0,001- 0,007 mSv). The yearly natural background dose in the Netherlands is 1000-2000 microSievert (1-2 mSv). The radiation risk is comparable with a X-ray in the dentist clinic.

6.3.6 Blood sample

For attenuation of the FDG-PET-CT-scan a catheter is necessary to inject the FDG. When the catheter is placed 10mL of blood is withdrawn for analysis of fasting glucose, catecholamines, free fatty acids, insulin, glycerol and thyroid gland function. The volume of 10mL will also be sufficient to save one sample for optional additional analyses, which will be possible after informed consent of the subject as signed for separately in the informed consent form.

6.4 Withdrawal of individual subjects

Subjects can leave the study at any time for any reason if they wish to do so without any consequences. The investigator can decide to withdraw a subject from the study for urgent medical reasons.

7. RISKS OF STUDY PROTOCOL

7.1 Risks of FDG-PET-CT and DXA scan

The advanced TF Gemini PET-CT scanner is equipped with time of flight electronics, which allows the use of a relatively low amount of radioactivity (2 mCi or 74MBq). The resulting total radiation dose from the low-dose CT scan and the injected radioactive tracer is 2.8 mSv. The effective radiation dose of the DXA scan is, depending on the duration of the scan (size of the body), between 1-7 *micro*Sievert (0,001- 0,007 mSv).
Together, this is considered as a low risk,[23] comparable with the yearly natural background dose in the Netherlands (1-2 mSv). Since each participant will undergo two scans, this means that the risk is comparable with the natural background radiation received in two years. The effective dose range (1-10 mSv) does not change, neither the risk category (<10 E-4) (see earlier MEC 07-3-056, amendment No. 2).
The clinical treatment program requires a I^{124} PET-CT scan (20Mbq x 9.1 mSv/MBq = 182mSv) for determination of thyroid gland remnants after thyroid gland resection and a I^{131} ablation therapy (2775MBq x 15mSv/MBq= 41625 mSv) of possible thyroid gland remnants. The additional radiation dose from the FDG-PET-CT- and DXA-measurements in this study is considered low in comparison to the standard radiation dose given for regular therapy.

8. SAFETY REPORTING

8.1 Section 10 WMO event

In accordance to section 10, subsection 1, of the WMO, the investigator will inform the subjects and the reviewing accredited METC if anything occurs, on the basis of which it appears that the disadvantages of participation may be significantly greater than was foreseen in the research proposal. The study will be suspended pending further review by the accredited METC, except insofar as suspension would jeopardize the subjects' health. The investigator will take care that all subjects are kept informed.

8.2 Adverse and serious adverse events

Adverse events are defined as any undesirable experience occurring to a subject during the study, whether or not considered related to the treatment. All adverse events reported spontaneously by the subject or observed by the investigator or his staff will be recorded.

A serious adverse event is any untoward medical occurrence or effect that at any dose:

- results in death;

- is life threatening (at the time of the event);
- requires hospitalization or prolongation of existing inpatients' hospitalization;
- results in persistent or significant disability or incapacity;
- is a congenital anomaly or birth defect;
- is a new event of the trial likely to affect the safety of the subjects, such as an unexpected outcome of an adverse reaction, lack of efficacy of an IMP used for the treatment of a life threatening disease, major safety finding from a newly completed animal study, etc.

All SAEs will be reported through the web portal ToetsingOnline to the accredited METC that approved the protocol, within 15 days after the sponsor has first knowledge of the serious adverse reactions.
SAEs that result in death or are life threatening should be reported expedited. The expedited reporting will occur not later than 7 days after the responsible investigator has first knowledge of the adverse reaction. This is for a preliminary report with another 8 days for completion of the report.

8.3 Follow-up of adverse events

All adverse events will be followed until they have abated, or until a stable situation has been reached. Depending on the event, follow up may require additional tests or medical procedures as indicated, and/or referral to the general physician or a medical specialist.

9. STATISTICAL ANALYSIS

Data analysis will be performed using PASW Statistics 18.0 for Mac OS X.
Statistical significance will be set at $p<0.05$.

9.1 Descriptive statistics

Data will be expressed as means±standard deviations of the mean.

9.2 Univariate analysis

To assess the effect of thyroid hormone levels on BAT activity both parameters will be tested for correlation using Pearson’s correlation coefficient. Between both analyses, a comparison will be made within subjects for energy expenditure, body composition (body weight, body fat), core and skin temperature, skin perfusion and thyroid function. This will be tested with a dependent paired samples t-test. Statistical significance will be set at $p<0.05$.

9.3 Multivariate analysis

Linear regression analyses will be used to identify correlations between primary and secondary outcome parameters.

10. ETHICAL CONSIDERATIONS

10.1 Regulation statement

The study will be conducted according to the principles of the Declaration of Helsinki and in accordance with the Medical Research Involving Human Subjects Act (WMO).[24]

10.2 Recruitment and consent

With the results of this investigation, we hope to gain further insight into the development and treatment of obesity due to the exploration of the function of BAT in the human body. The study has to be approved by the Medical Ethical Committee of Maastricht University. The very specific population in this study will be recruited from the clinical patient cohort undergoing total thyroid resection for well-differentiated thyroid carcinoma, without any preselection (except children and mentally disabled patients). An information letter is handed or sent to this population. After subjects are given 2 weeks time for reflection they are called to inform if they are interested to participate in this study. If so, an informed consent is obtained. If not, the subject is not contacted anymore regarding the proposed study.

There can be a time window between the visit in the outpatient clinic and the actual moment of surgery and subsequent withdrawal/rTSH therapy. Therefore, after inclusion as described above the subject will be re-contacted by telephone by the researcher two weeks before the study measurements are performed (2-4 weeks after surgery). At this moment, the subject will be asked to re-confirm informed consent to be sure the subject still wants to participate in the study. In addition, the physician (Dr. B. Havekes or Dr. M. Kars) will need to confirm that the subject is in a stable medical and psychological condition to be able to participate in the study. If the subject does not want to participate or is in an unstable condition (as judged by the treating specialist), the contact with the researcher ends. If the patient wants to participate, both measurements are planned by the researcher in accordance with the clinical treatment program.

An independent MD is assigned to this project. Subjects are free to withdraw at any stage of the study without giving a reason.

The measurements that are performed can show unexpected findings. For example, PET-CT can reveal an unknown malignancy. Subjects are informed that these findings can be found in the measurements performed in this study. The subject and his general practitioner will be informed about these findings when they occur. If the subject does not want to be informed about unexpected findings, inclusion will not be possible.

10.3 Compensation for injury

Maastricht University has a liability assurance for studies with human subjects. Maastricht University also has an insurance that provides cover for damage to research subjects through injury or death caused by the study. Both are in accordance with the legal requirements in the Netherlands (Article 7 WMO and the Measure regarding Compulsory Insurance for Clinical Research in Humans of 23th June 2003).

- € 450.000,-- (i.e. four hundred and fifty thousand Euro) for death or injury for each subject who participates in the Research;
- € 3.500.000,-- (i.e. three million five hundred thousand Euro) for death or injury for all subjects who participate in the Research;
- € 5.000.000,-- (i.e. five million Euro) for the total damage incurred by the organisation for all damage disclosed by scientific research for the Sponsor as 'verrichter' in the meaning of said Act in each year of insurance coverage.

The insurance applies to the damage that becomes apparent during the study or within 4 years after the end of the study. The Maastricht University has a third-party insurance.

11. ADMINISTRATIVE ASPECTS AND PUBLICATION

11.1 Handling and storage of data and documents

At the start of the study, subjects will be assigned a random number that will not change during the study. This number is linked with the name, address, date of birth, and telephone number of the subject in a password protected file. Only members of the project team can access this file. The random number will be used for subject identification.

In the informed consent form the subject's permission is asked to store blood samples withdrawn in this study with a maximum of 15 years.

Also, the subject's permission is asked to store these blood samples with a maximum of 15 years to perform additional analyses in the scope of this research. The scope of this proposal is BAT physiology, obesity and energy metabolism.

In case data or documents will be analyzed outside the scope of this proposed study, the subjects will need to be asked for permission. When data collected in this study can be used for future research in the scope of this study, no permission will be asked. This concerns data from the PET-CT-scans and blood samples.

11.2 Amendments

All amendments will be notified to the METC that gave a favourable opinion.

All substantial amendments will be notified to the METC and to the competent authority.

Non-substantial amendments will not be notified to the accredited METC and the competent authority, but will be recorded and filed.

11.3 Annual progress report

The investigator will submit a summary of the progress of the trial to the accredited METC once a year. Information will be provided on the date of inclusion of the first subject, numbers of subjects included and numbers of subjects that have completed the trial, serious adverse events/ serious adverse reactions, other problems, and amendments.

11.4 End of study report

The investigator will notify the accredited METC of the end of the study within a period of 8 weeks. In case the study is ended prematurely, the investigator will notify the accredited METC, including the reasons for the premature termination.

Within one year after the end of the study, the investigator/sponsor will submit a final study report with the results of the study, including any publications/abstracts of the study, to the accredited METC.

11.5 Public disclosure and publication policy

The results of this study will be disclosed unreservedly in scientific journals. The participants are entitled to public disclosure of the results of the study.

12. REFERENCES

1. Nedergaard J, Bengtsson T, Cannon B. Unexpected evidence for active brown adipose tissue in adult humans. Am J Physiol Endocrinol Metab 2007;293:E444-52.
2. van Marken Lichtenbelt WD, Vanhommerig JW, Smulders NM, et al. Cold-activated brown adipose tissue in healthy men. N Engl J Med 2009;360:1500-8.
3. Virtanen KA, Lidell ME, Orava J, et al. Functional brown adipose tissue in healthy adults. N Engl J Med 2009;360:1518-25.
4. Cannon B, Nedergaard J. Brown adipose tissue: function and physiological significance. Physiol Rev 2004;84:277-359.
5. Tseng YH, Cypess AM, Kahn CR. Cellular bioenergetics as a target for obesity therapy. Nat Rev Drug Discov 2010;9:465-82.
6. Nedergaard J, Bengtsson T, Cannon B. New powers of brown fat: fighting the metabolic syndrome. Cell Metab 2011;13:238-40.
7. Vijgen GH, Bouvy ND, Teule GJ, Brans B, Schrauwen P, van Marken Lichtenbelt WD. Brown adipose tissue in morbidly obese subjects. Plos One 2011;6:e17247.
8. Bianco AC, Salvatore D, Gereben B, Berry MJ, Larsen PR. Biochemistry, cellular and molecular biology, and physiological roles of the iodothyronine selenodeiodinases. Endocr Rev 2002;23:38-89.
9. Lee JY, Takahashi N, Yasubuchi M, et al. Triiodothyronine Induces UCP1 Expression and Mitochondrial Biogenesis in Human Adipocytes. Am J Physiol Cell Physiol 2011.
10. Bianco AC, Silva JE. Intracellular conversion of thyroxine to triiodothyronine is required for the optimal thermogenic function of brown adipose tissue. J Clin Invest 1987;79:295-300.
11. de Jesus LA, Carvalho SD, Ribeiro MO, et al. The type 2 iodothyronine deiodinase is essential for adaptive thermogenesis in brown adipose tissue. J Clin Invest 2001;108:1379-85.
12. Endo T, Kobayashi T. Thyroid-stimulating hormone receptor in brown adipose tissue is involved in the regulation of thermogenesis. Am J Physiol Endocrinol Metab 2008;295:E514-8.
13. Castillo M, Hall JA, Correa-Medina M, et al. Disruption of thyroid hormone activation in type 2 deiodinase knockout mice causes obesity with glucose intolerance and liver steatosis only at thermoneutrality. Diabetes 2011;60:1082-9.
14. Lopez M, Varela L, Vazquez MJ, et al. Hypothalamic AMPK and fatty acid metabolism mediate thyroid regulation of energy balance. Nat Med 2010;16:1001-8.
15. Skarulis MC, Celi FS, Mueller E, et al. Thyroid hormone induced brown adipose tissue and amelioration of diabetes in a patient with extreme insulin resistance. J Clin Endocrinol Metab 2010;95:256-62.
16. Mentuccia D, Proietti-Pannunzi L, Tanner K, et al. Association between a novel variant of the human type 2 deiodinase gene Thr92Ala and insulin resistance: evidence of interaction with the Trp64Arg variant of the beta-3-adrenergic receptor. Diabetes 2002;51:880-3.
17. Cannon B, Nedergaard J. Thyroid hormones: igniting brown fat via the brain. Nat Med 2010;16:965-7.
18. van Marken Lichtenbelt WD, Daanen HAM, Wouters L, et al. Evaluation of wireless determination of skin temperature using iButtons. Physiol & Behav 2006;88:489-97.
19. Schoffelen PFM, Westerterp KR, Saris WHM, Hoor Ft. A dual-respiration chamber system with automated calibration. J Appl Physiol 1997;83:2064-72.
20. Marken Lichtenbelt W.D. van, Daanen H.A.M., Wouters L., et al. Evaluation of wireless determination of skin temperature using iButtons. Physiol & Behav 2006;88:489-97.
21. Parson KC. Human thermal environments. 2nd ed. London: Taylor&Francis; 2003.
22. van Marken Lichtenbelt WD, Hartgens F, Vollaard NBJ, Ebbing S, Kuipers H. Body composition changes in bodybuilders: a method comparison. Med Sci Sports Exerc 2004;36:490-7.

23. ICRP. Annals of the ICRP. Risks associated with ionising radiations: Pergamon Press; 1992.
24. Williams JR. The Declaration of Helsinki and public health. Bull World Health Organ 2008;86:650-2.
